# Supplementary material for: Whole genome sequencing refines stratification and therapy of patients with clear cell renal cell carcinoma
Source: Nat Commun. 2024 Jul 15;15:5935. doi: 10.1038/s41467-024-49692-1 (PMC11250826; doi:10.1038/s41467-024-49692-1)
Supplement: Supplementary file 1 — Supplementary Information [file 41467_2024_49692_MOESM1_ESM.pdf]

SUPPLEMENTARY METHODS

1. Sample collection 3
2. Whole genome sequencing and somatic variant calling 3
2.1. Genome sequencing and alignment 3
2.2. Single nucleotide variant and indel calling 4
2.3. Removing alignment bias introduced by semi-aligned-read soft-clipping 5
2.4. Evaluating microsatellite instability 5
2.5. Copy number alteration profiling 5
2.5.1. Stage 1: Initial profiling of copy number alterations 5
2.5.2. Stage 2: Evaluation of profile concordance with variant allele frequency distributions 6
2.5.3. Stage 3: Quality assessment 7
2.5.4. Stage 4: Re-profiling of copy number alterations 8
2.6. Structural variant calling 8
2.7. Whole genome duplication classification 9
2.8. Estimation of telomere length 9
3. Clinical data 9
4. Sample selection 10
5. Single nucleotide variant and indel drivers 11
5.1. Variant Effect Prediction 11
5.2. Identification of coding drivers 12
5.2.1. Input mutation pre-processing 12
5.2.2. Identification of coding drivers 12
5.3. Functional annotation of drivers and clinical actionability 14
5.4. Pathways 16
5.5. Non-coding drivers 16
6. Somatic copy number alteration patterns 17
6.1. Copy number alteration classification 17
6.2. Enrichment of copy number alterations 17
6.2.1. Preparing GISTIC input and Initialization 17
6.2.2. Prioritising likely gene targets of focal amplifications and deletions 18
7. Somatic structural variation patterns 19
7.1. Classification of simple and complex structural variants 19
7.2. Simple structural variation hotspots 20
7.2.1. Evaluating relationships between genomic features and SV rates 20
7.2.2. Simulating SVs 21
7.2.3. Identifying SV hotspots 22
7.3. Classification of SV hotspots as fragile sites 22
8. Mutational Processes 23
8.1. Characterising single-base-substitution, doublet-base-substitution and indel signatures 23
8.2. Predicting homologous recombination deficiency 24
9. Mutation Timing 24
9.1 Timing of copy number alterations and somatic mutations 24

9.2 Relative ordering of driver events 24
10. Immune Profiling 25
10.1. Human Leukocyte Antigen (HLA) Typing 25
10.2. Neoantigen Prediction 26
11. Correlating Clinicopathological and Mutational Variables 27
11.1. Correlations with Mutational Attributes 27
11.2. Survival Analysis 28

Source of each software package and external downloaded data is shown in Supplementary Table 24.

1. Sample collection

Tumour and germline sequencing data were obtained from the main programme version 14 release of the 100,000 Genomes Project (100kGP), an NHS initiative for high-throughput cancer sequencing[1-2]. The recruitment of patients was organised by 13 Genomic Medicine Centres (GMCs) and affiliated hospitals from across the UK. The renal tumour cases included in this study were all routine surgical cases reported by diagnostic histopathologists at contributing centres. Histology of RCC was as per WHO Classification of Urinary and Male Genital tumours 3rd/4th edition[3], which have matching clear cell renal cell carcinoma (ccRCC) diagnostic criteria. Written informed consent was provided by all patients. The collection of tissue and the preparation, extraction and quantification of DNA was undertaken locally, followed by transfer of DNA to a central biorepository. Illumina conducted whole genome sequencing of paired tumour/normal DNA. Processed BAM files were sent to Genomics England, who performed additional quality checks and managed data storage.

2. Whole genome sequencing and somatic variant calling

2.1. Genome sequencing and alignment

Sequencing data for primary ccRCC samples were obtained from the 100kGP Renal Cancer Domain main programme version v14 release. Sample preparation was conducted using Illumina TruSeq DNA PCR-free library preparation kit and sequencing completed using HiSeq X, generating 150 base pair (bp) paired-end reads. We restricted our analysis to WGS data generated on fresh frozen and PCR-free samples to ensure low-input fast workflow, uniform coverage and high sequencing accuracy (*i.e* avoiding bias artefacts introduced due to PCR duplicates, especially in GC rich regions). Normal blood and tumour samples were sequenced to average depths of 30x and 100x respectively. Samples with poor sequencing quality were identified and removed using principal components analysis, considering (1) AT/CG dropout, (2) percentage of mapped reads, (3) percentage of chimeric DNA fragments, (4) average insert size and (5) local coverage unevenness. Samples of poor sequencing quality were not included in the 100kGP main programme v14 release and were therefore not considered in any of our analyses. Initial sequencing analysis was conducted using Illumina's North Star pipeline (version 2.6.53.23). Sequence alignment to the *Homo sapiens* GRCh38Decoy assembly was completed using Isaac (version 03.16.02.19)[4].

2.2. Single nucleotide variant and indel calling

Single nucleotide variants (SNV) and small insertions/deletions (indels) were called using Strelka[5] (version 2.4.7). Additional filters added alongside the default Strelka filters to remove variants include:

- Variants with established high population germline allele frequency (≥1%) according to the gnomAD dataset[6] and/or the 100kGP dataset.
- Variants with excessive somatic frequency (≥5%) in cancer according to the 100kGP dataset. The 5% threshold was based on the frequency of recurrent non-synonymous variants in hallmark genes of the Cancer Gene Census[7].
- Variants labelled as simple repeats as classified by Tandem Repeats Finder[8].
- Indels for which ≥10% of base calls in a window of 50 bases on each side of the indel have been filtered by Strelka because of high sequencing noise.
- If the majority of overlapping 150bp reads are reads that map to multiple loci, then the reads involved in the overlap are removed. This step was ignored when assessing single base substitution signatures (see Section 8).
- SNVs which had their respective ratio of tumour allele depths showing statistical evidence of being different to the ratio of allele depths (via Fisher's exact test) at this site in a panel of normal samples (PoN). Only individuals not carrying the relevant alternate allele at a particular site were included in the allele depth counts. The PoN consisted of 7,000 non-tumour genomes from the 100kGP, and the bcftools mpileup function (version 1.9) was used to count PoN allele depths. To ensure similarity to the Strelka preset filters, duplicate reads were removed and quality thresholds set at base quality ≥5, mapping quality ≥5, and phred score <80 (Fisher's exact test). The phred score threshold was chosen based on optimising the precision and recall from a TRACERx truth set[9].

2.3. Removing alignment bias introduced by semi-aligned-read soft-clipping

The soft clipping of semi-aligned reads by Isaac results in the loss of support for alternate alleles occurring within five bases of each read end. We used FixVAF to remove allelic bias introduced by this soft clipping[10]. FixVAF soft clips all reads by five bases at each end, irrespective of whether any of the bases are variant sites

and whether the reads support a reference or alternate allele. Reads supporting small insertions and deletions at the variant position are ignored.

2.4. Evaluating microsatellite instability

mSINGS was used to identify tumours with evidence of microsatellite instability (MSI)[11]. Background models were generated following the previously described procedure (https://github.com/sheenamt/msings/blob/master/Recommendations_for_custom_assays). MISA was used to generate microsatellite sites[12] and sites were only considered if they overlapped regions of good mappability. Sites were also removed if they were measured as unstable in >5 microsatellite stable (MSS) test tumours or not unstable in >0 test MSI tumours. After validation of the background model, mSINGS was run on the ccRCC tumour samples.

2.5. Copy number alteration profiling

Clonal and subclonal copy number alterations (CNAs) were called using a four-stage iterative procedure incorporating Battenberg v2.2.8[13] (Supplementary Fig. 16 - 17):

2.5.1. Stage 1: Initial profiling of copy number alterations

Battenberg was used to detect clonal and subclonal CNAs and to estimate sample purity and tumour ploidy[13]. Briefly, numbers of reads supporting SNV reference and alternate alleles were counted in tumour and normal samples. Heterozygous SNPs were phased using SHAPEIT2 v2.r904[14] and A and B alleles were assigned. These data were then segmented using piece-wise constant fitting[15] and subclonal copy number segments identified using t-tests. Sample purity and tumour ploidy were estimated using the method described by Van Loo, *et al.* [16]. As sequencing data were aligned to hg38, SNP positions were converted to hg37 before phasing, and output segments were converted back to hg38.

2.5.2. Stage 2: Evaluation of profile concordance with variant allele frequency distributions

Expected variant allele frequency is dependent on the fraction of tumour cells containing the variant, the tumour copy number profile, the number of chromosome copies with the variant (multiplicity) and the sample purity[17]. Given the tumour copy number profile and an estimated sample purity, we can therefore

expect to observe enrichment of variants with allele frequencies approximating particular values (representing variants present in all tumour cells)[16]. Failure to observe such enrichment would suggest that either the copy number profile or sample purity is incorrect, and we therefore assessed Battenberg output validity via the SNV variant allele frequency (VAF) distributions.

When evaluating SNV VAF distributions, only autosomal genome segments with copy number states of 1:1, 1:0, 2:2, 2:1, and 2:0, with no evidence of subclonal copy number changes, were considered. Each of these five copy number states was evaluated separately, as the possible variant multiplicities and expected clonal SNV VAFs differ between them[16]. Copy number states corresponding to genome regions containing <5% of all SNVs were not considered. Expected locations of VAF distribution peaks were computed as:

$$\frac{\rho_{Battenberg} m}{2(1-\rho_{Battenberg})+\rho_{Battenberg}\psi_v} \quad (1)$$

Where $\rho_{Battenberg}$ is the sample purity estimated by Battenberg, ψ_v the ploidy of the tumour at the variant site, and m the variant multiplicity, which can equal 1 or 2 in copy number states of 2:2, 2:1 and 2:0, and only 1 in states of 1:1 and 1:0. VAF distribution peaks were called using kernel density estimation, implemented in peakPick v0.11[18], with peaks corresponding to densities <0.3 being excluded. For each copy number state, the expected peak location corresponding to the highest considered variant multiplicity was matched to the observed VAF distribution peak with the greatest VAF, whilst any other expected peak location was matched to the observed peak with the most similar VAF. Tumour heterogeneity can prevent VAF peak detection, and therefore for samples where ≥1 expected peak locations were considered, the expected peak furthest from the respective matched observed peak (in terms of VAF) was discarded. Sample purity (ρ_i) was re-estimated for each remaining expected peak location (with VAF a) using the matched observed peak VAF:

$$\rho_i = \frac{2a}{m+\omega(2-\psi_s)} \quad (2)$$

Where ω is the VAF of the matched observed peak and ψ_s the ploidy of the respective copy number state. Greater variant numbers improve our ability to call peaks, and therefore a single new purity estimate (ρ_{new}) was computed as the weighted average of the peak-wise purity estimates (and used when re-profiling samples):

$$\rho_{new} = \sum_i \frac{n_i \rho_i}{N q_i} \quad (3)$$

Where n_i is the number of SNVs in genome regions of the respective copy number state, *N* is the number of SNVs in genome regions of all considered copy number states, and q_i is the number of considered variant multiplicities for the respective copy number state. When assessing CNA profile quality, the weighted average of the difference between the purity estimated by Battenberg and the peak-wise purity estimates was used:

$$\eta = \sum_i \frac{n_i|\rho_i - \rho_{Battenberg}|}{Nq_i} \quad (4)$$

2.5.3. Stage 3: Quality assessment

Multiple criteria were used to assess Battenberg output validity:

- VAF distribution peaks were observed at the correct locations (defined as η <5%).
- DPClust[13] identified a clonal mutation cluster (defined as a cluster containing ≥5% of all SNVs with a CCF of between 0.9 and 1.1).
- DPClust identified no "super-clonal" mutation clusters (defined as clusters containing ≥5% of all SNVs with CCFs >1.1).
- Where Battenberg identifies most of the genome to be tetraploid (2:2), a peak in the SNV VAF distribution in 2:2 regions corresponding to a variant multiplicity of 1 is observed.
- No single homozygous deletion >10Mb is called.

Samples satisfying all criteria were deemed to pass and their CNA profiles and purity estimates were used in subsequent analyses. Samples not satisfying at least one criterion were deemed to fail and were re-profiled (*i.e.* proceeded to stage 4).

2.5.4. Stage 4: Re-profiling of copy number alterations

Samples failing quality assessments were re-profiled a maximum of three times using Battenberg with new purity and ploidy estimates. Samples failing quality assessment after three re-profiling attempts were not considered in subsequent analyses. The new purity (ρ_{new}) was estimated in stage 2, whilst the new ploidy (ψ_{new}) was estimated using the method described by Van Loo, *et al.*[16]:

$$\psi_{new} = \frac{\rho_{Battenberg}\,(\psi_{Battenberg} - 2) + 2\rho_{new}}{\rho_{new}} \quad (5)$$

2.6. Structural variant calling

Somatic structural variants (SV) were called using a graph-based consensus approach using Delly[19], Lumpy[20], Manta[21] and considering support from copy number alterations. SVs were first called using the three individual SV callers with default parameters. Delly was run with post-filtering of somatic SVs using all normal samples, as described in the Delly documentation. SVs from the three individual callers were further filtered if any reads supporting the variant were identified in the matched normal, if <2% of tumour reads supported the variant, or if either variant breakpoint was located in a telomeric or centromeric region or on a non-standard reference contig (*i.e.,* not chromosomes 1-22, X or Y). Remaining SVs were merged with a modified version of PCAWG Merge SV, which uses a graph-based approach to identify and merge SVs identified by multiple callers, allowing a 400bp slot for the breakpoint positions[22]. SVs were included in the final SV data set if they were identified by at least two SVs callers, or by a single SV caller but with a breakpoint within 3kb of a called CNA segment boundary (Supplementary Fig. 18).

Somatically acquired long interspersed nuclear element (LINE-1) retrotransposition events were identified using xTea[23]. Alu elements, SINE-VNTR-Alu elements and processed pseudogenes were not called as together they comprise ≤3% of cancer retrotransposition events[24]. Retrotransposition events are mechanistically distinct from other SV-generating events[24] and we therefore didn't consider retrotransposition events in our SV analyses. SVs called by the graph-based consensus approach were categorised as likely retrotransposition events and excluded if: (i) xTea identified a transduced region in the same tumour sample within 10kb of either rearrangement break point, or (ii) xTea identified a transduced region within 10kb of either rearrangement breakpoint in ≥1% renal tumour samples. A 10kb threshold was used as most somatically acquired transductions span regions <10kb from a LINE-1 element[25].

2.7. Whole genome duplication classification

The average genome copy number state (ψ_{ave}) was calculated to define the threshold used to classify each tumour as whole genome duplicated (WGD):

$$\psi_{ave} = \frac{\sum_{i=1}^{S}(L_i \sum_{j=1}^{2}(F_{j,i}(C_{j,i}^{Maj}+C_{j,i}^{Min})))}{\sum_{i=1}^{S} L_i} \quad (6)$$

Where S is the number of copy number genome segments, $F_{j,i}$ is the fraction of tumour cells carrying copy number state j for genome segment i, $C_{j,i}^{Maj}$ and $C_{j,i}^{Min}$ are the major and minor allele copy numbers for state

j for genome segment i, and L_i is the base pair length of genome segment i. If there is no evidence of a subclonal alteration, then $F_{1,i} = 1$ and $F_{2,i} = 0$. WGD classification is then made by (Gerstung, *et al.*[26]):

$$\{WGD,\ if\ 2.9 - 2H < \psi_{ave}; Not\ WGD,\ otherwise\} \quad (7)$$

Where H is the fraction of the respective genome where loss of heterozygosity occurred (minor allele copy number = 0).

2.8. Estimation of telomere length

Tumour and germline telomere lengths were estimated from their respective bam files using TelomereCat (v3.3.0)[27] with default parameters applied.

3. Clinical data

Demographic and clinical data were obtained from the Genomic Medicine Centers (GMC; https://www.england.nhs.uk/genomics/nhs-genomic-med-service/), NHS Digital (NHSD; https://digital.nhs.uk/), Public Health England's National Cancer Registration and Analysis Service (PHE-NCRAS; https://www.gov.uk/guidance/national-cancer-registration-and-analysis-service-ncras) and tumour pathology reports, through the Genomics England Research Environment. Sequenced tumour samples were matched to their corresponding PHE-NCRAS records using the reported tumour sampling and PHE-NCRAS treatment dates, allowing a seven-day maximum discrepancy.

All samples had data consisting of sex, date of cancer diagnosis, date of tumour sampling, age at tumour sampling, date of last reported clinical follow-up, survival outcome and date of death if applicable, tumour grade, and tumour stage.

For some variables, data were obtained from multiple sources (GMC, NHSD, PHE-NCRAS and curated pathology reports). Potential conflicts between these data sources were reviewed manually, with information from curated pathology reports taking precedence. For 202 of the patients, information on progression free survival (PFS) and treatment response was collected by directly liaising with GMCs and participating clinicians. Progression free-survival (defined as the survival time without disease progression

on current systemic treatment for ≥ 6 months), with disease progression being defined as per RECIST 1.1 (*i.e.* at least a 20% increase in the sum of diameters of target lesions).

4. Sample selection

949 fresh-frozen PCR-free ccRCC primary tumour samples from the 100kGP Renal Cancer Domain main programme version 14 release were studied. Tumour samples were subjected to the following clinical data quality control (QC) procedures, although no samples failed these criteria (Supplementary Table 23):

- Age at tumour sampling was not available.
- The participant was <18 years old at the time of tumour sampling.
- Sex inferred from sequencing data did not match the sex reported by the submitting GMC.

Tumour sample purity and sequencing data quality affect the sensitivity and precision of variant calling[5] and we therefore excluded samples using the following QC procedures. A total of 59/949 sequenced tumour samples were excluded because of these criteria (Supplementary Table 23):

- Tumour samples were excluded if sample cross-contamination was >1%.
- Tumour samples were excluded if the cross-contamination of the matched germline sample with another germline sample was >1%.
- Estimating exact tumour sample purity is difficult if purity is low[28] and we therefore also excluded those tumour samples in which the majority of called SNVs had low VAFs, as this can occur due to low sample purity[17]. Tumour samples with a median SNV VAF <0.1 were therefore excluded, with the threshold chosen based on the smaller numbers of potential driver variants called in ccRCC samples.
- Tumour samples were excluded if <500 SNVs were called, as this number is below the smallest number of SNVs previously reported in ccRCC in PCAWG[29] and therefore suggestive of low sample purity or sequencing data quality.

CNA profiles passing all CNA QC criteria could not be generated for 75/949 sequenced tumour samples, necessitating their exclusion. Some participants had multiple tumour samples sequenced, representing 67/949 tumour-normal pairs. For each multi-sample participant we therefore included only the tumour sample with the highest purity in the primary analysis. This resulted in a cohort of 778 primary ccRCC tumour samples, all from unique participants (Supplementary Table 1).

5. Single nucleotide variant and indel drivers

5.1. Variant Effect Prediction

To run driver-identification programmes, all somatic mutations were first annotated using Variant Effect Predictor (VEP) built on Ensembl (v101, Grch38, McLaren *et al.*[30]). Each VCF was annotated using the commands: *vep -i --assembly GRCh38 –no_stats –cache –offline –symbol –protein -o --vcf –canonical –dir --hgvs –hgvsg –fasta --plugin CADD, --plugin UTRannotator*.

The VEP Combined Annotation Dependent Depletion (CADD, v1.6, https://github.com/kircherlab/CADD-scripts[31–33]) plugin was applied the CADD score file for all SNV and indel mutations. Five prime untranslated regions (5' UTR) were annotated using the "UTRannotator" plugin (Zhang *et al.*[34]). Certain mutations were also mapped to their canonical protein-coding transcripts from Ensembl. We define non-synonymous mutations as any SNV or indel classified as having a moderate or high calculated consequence as stated by VEP.

5.2. Identification of coding drivers

The Integrative OncoGenomics pipeline (IntOGen[35], downloaded February 2021) was used to identify an initial set of candidate protein-coding driver genes (Section 5.2.2). An additional filtering step was applied to remove false positives (Section 5.2.3).

5.2.1 Input mutation pre-processing

Tumour samples were flagged for exclusion from driver gene identification if they contained either >10,000 mutations, representing hypermutated status, or had an outlier mutation count compared to the rest of the cohort, defined as upper quartile + 1.5 × interquartile range. This led to 16/778 tumour samples being excluded from the downstream driver analysis. Mutations in the Hartwig Consortium (PoN) were also excluded[36].

5.2.3 Identification of coding drivers

Seven driver gene identification methods were applied, with each one prioritising different properties of the sample mutational profile when selecting candidate driver genes.

1. dNdSCV[37] selects genes under positive selection based on an excess of nonsynonymous (missense, nonsense, essential splice) mutations compared with synonymous mutations (after correction for local trinucleotide context) on a gene.
2. OncodriveFML[38] detects driver genes showing an enrichment of mutations with higher functional impact than expected (based on Cadd Scores[31–33]; Section 5.1).
3. OncodriveCLUSTL[39] selects driver genes containing clusters that are enriched with larger numbers of mutations than expected compared to a simulated nucleotide context-based background model.
4. cBaSE[40] detects driver genes under positive selection based on per-gene probabilities of missense and nonsense mutations, which are compared to a simulated neutral mutation model.
5. MutPanning[41] detects driver genes exhibiting an enrichment of mutations with sequence contexts that deviate from the contexts of their surrounding mutations more than expected.
6. HotMaps3D[42] evaluates if a driver gene has a hotspot of missense mutations in the 3-dimensional protein structure. Protein structures are from The Protein Data Bank[43] (PDB, downloaded March 2020).
7. smRegions[44] detects higher enrichment of nonsynonymous mutations than expected in gene regions of interest such as protein domains. This analysis utilised information from protein family (pfam) domains which were mapped to Ensembl[45] (v101) canonical transcripts.

IntOGen's weighted ranking system that combines the seven driver gene identification methods was applied. First the top-40 ranked genes and their corresponding *P*-values in each of the seven driver identification methods were collected. A truth set of known driver genes was created from any Tier 1 or Tier 2 somatically mutated genes in the COSMIC Cancer Gene Census[46] (CGC). Lists of per-method ranks were combined using Schulze's voting method to generate a single "consensus" ranking that takes into account how well each method gives precedence to the top genes in said truth set, with corresponding *P*-values combined using a weighted Stouffer *Z*-score. Finally, multiple test correction was applied to create two sets of *Q*-values, one set containing all genes and the other considering CGC genes only. Driver candidates were then assigned the following tiers based on the sets of ranks:

- Tier 1 candidate drivers are high confidence drivers defined by having a Schulze's voting consensus ranking higher than the consensus ranking of the first gene with a corresponding Stouffer $Q > 0.05$.
- Tier 2 candidate drivers are medium confidence drivers, which are CGC genes and show a Stouffer CGC $Q < 0.25$, but lack the consensus ranking criteria of tier 1 genes.
- Tier 3 represents lower confidence candidate drivers, which could still be rescued through post processing, that have a Stouffer $Q < 0.05$ but are not Tier 1 or 2 CGC drivers.

- Tier 4 represents candidate genes that do not satisfy Tier 1 or Tier 2 criteria, with Stouffer Q >0.05. These candidates are thus unlikely to be drivers.

In addition to the IntOGEN ranking, candidate driver genes were filtered based on the following annotations:

1) AUTOMATIC FAIL. A candidate driver gene would be excluded from further consideration if annotated with at least one of the following:
   a) TIER4. Categorised as tier 4.
   b) 1_METHOD. Only significant (Q <0.1) in one method.
   c) EXPRESSION. Gene reported as having very low or no expression in KIRC TCGA data.
   d) OLFACTORY_RECEPTOR. Gene is an olfactory receptor gene.
   e) KNOWN_ARTIFACT. Gene is a known artefact or long gene (e.g., TTN).
   f) STRAND BIAS. Gene has a nonsynonymous SNV with corresponding strand bias ≥10 (Strelka Info field SNVSB).
2) MANUAL REVIEW. If a gene is not excluded based on any AUTOMATIC FAIL filters, it is retained as a candidate driver:
   a) GERMLINE. Non-tier 1 CGC gene has ≥1 mutations per sample and oe_syn/ms/lof > 1.5 based on GnomAD (v2.1) constraint metric estimates.
   b) SAMPLE_3_MUTS. Non-CGC gene where there are ≥3 mutations in ≥1 tumour.
   c) LITERATURE. Non-CGC gene where there are no literature annotations according to CancerMine[47].
3) AUTOMATIC PASS. Is not flagged by any AUTOMATIC FAIL or MANUAL REVIEW filters.

Candidate driver roles were assigned using dN/dS ratios for missense (w_{mis}) and nonsense (w_{non}) mutations for the given gene derived from dNdSCV (https://bitbucket.org/intogen/intogenplus/src/master/core/intogen_core/postprocess/drivers/role.py):

- A "distance" metric was calculated by $distance = \frac{(w_{mis} - w_{non})}{\sqrt{2}}$
- Candidate drivers with distance >0.1 represent those with an excess of missense to nonsense mutations and are therefore considered oncogenes.
- Candidate drivers with distance <0.1 represent those with an excess of nonsense to missense mutations and are therefore considered tumour suppressor genes (TSGs).
- Otherwise, the role of the candidate driver is unclear and considered ambiguous.

Gene candidates were annotated by their overlap with any IntOGen cohorts from a previous IntOGen pan-cancer analysis (01 Feb 2020) as well as from a pan-cancer TCGA analysis[48]. The nonsynonymous mutation frequency in driver genes were compared with TCGA[49], TRACERx[50], PCAWG[29] and CPTAC[51].

5.3. Functional annotation of drivers and clinical actionability

To determine the functional basis of novel driver genes we considered gene perturbation screening data from DepMap[52,53]. Across 16 ccRCC cell lines, genes were knocked out using CRISPR loss-of-function screens and the gene effect measured. To complement this approach we also considered gene expression data from TCGA[54] and GTEx[55], accessed through GEPIA[56]. The log fold change between tumour and normal tissue using the median expression was calculated and the direction of change compared with the known or predicted role of the gene (TSG/oncogene). Genes were annotated as being directly relevant to the biology of ccRCC if both the altered gene effect in over 50% of the cell lines and gene expression change was concordant with the behaviour expected from a known/predicted TSG (increase in cell line fitness from knockout + loss of expression) or oncogene (decrease in cell line fitness from knockout + gain of expression).

To assess the clinical relevance of individual mutations, in addition to Ensembl Variant Effect Prediction (see Section 5.1), all unique missense SNVs were annotated using AlphaMissense[57] to compare pathogenicity between driver and non-driver mutations.

Nonsynonymous mutations were additionally annotated using the OncoKB API[58]. OncoKB (v3.11) contains a repository of 682 gene transcripts, such that mutations within 23/38 driver genes could be annotated (Supplementary Table 4). In the first instance, the HGVSg identifier was used. In the rare instances that this failed, a combination of gene symbol, consequence and HGVSp were used to map mutations to OncoKB annotations. Nonsynonymous mutations in candidate driver genes were annotated as oncogenic if any of the following criteria were met, and otherwise labelled as variants of uncertain significance (VUS):

1. The mutation is annotated by OncoKB as “Oncogenic”, “Likely Oncogenic” or “Predicted Oncogenic”.
2. The mutation is classified as missense and was recurrent (present in $\geq$3 tumours).
3. The mutation is within a TSG driver and is classified as a protein-truncating mutation (splice acceptor, splice donor, frameshift, stop lost, stop gained or start lost).

All annotated mutations were analysed to see if these mutation biomarkers had corresponding FDA-recognised drugs (Supplementary Table 7), based on OncoKB v3.11.

We further assessed the clinical actionability of driver gene mutations by interrogating OncoKB Knowledge Base[58] (version 3.11). We segregated treatments targeting OncoKB annotated mutations as: Level 1 - FDA-recognised biomarker predictive of response to an FDA-approved drug in this condition; Level 2 - Standard care biomarker recommended by the NCCN or other professional guidelines predictive of response to an FDA-approved drug in this indication; Level 3 - Compelling clinical evidence supporting the biomarker as being predictive of response to a drug in this indication/Standard care or investigational biomarker predictive of response to an FD-approved or investigational drug in another indication; Level 4 - Compelling biological evidence supporting the biomarker as predictive. We also examined the COSMIC Mutation Actionability in Precision Oncology database[7], with treatments targeting VEP annotated mutations defined as one of the following; Level 1 - Approved marketed drug with demonstrated efficacy at the mutation; Level 2 - Phase 2/3 clinical results meeting primary outcome measures; Level 3 - Drug in ongoing clinical trials; Level 4 - Case studies.

5.4. Pathways

Cellular pathways containing driver genes (Supplementary Table 4), identified from both the IntoGen and non-coding pipelines (considering core promoters, distal promoters, 3' UTR, 5' UTR and non-canonical splice site of drivers), involved in tumourigenesis were found by literature search using PubMed. Pathways were also further validated via ActivePathways[59] which searches across gene ontology gene sets, oncogenic signature gene sets, canonical pathway curated gene sets and Reactome pathway curated gene sets obtained from MSigDB[60,61] (v7.5.1).

5.5. Non-coding drivers

We searched for non-coding drivers within the following genomic regions referenced by Ensembl[45] (v101): (i) core promoters, <200bp downstream from the transcriptional start site (TSS) and <50bp upstream of the TSS of canonical protein-coding transcripts (n=19,283); (ii) distal promoters, <2kb upstream of the TSS of canonical protein-coding transcripts (n=19,296); (iii) 5'UTRs of canonical protein-coding transcripts (n=18,613); (iv) 3' UTRs of canonical protein-coding transcripts (n=18,806); (v) non-canonical splice regions being any region that extends 30bp into the intron from essential splice donor or acceptor sites of canonical protein-coding transcripts (n=18,163); (vi) LincRNAs (n=16,510). Candidate regions overlapping coding sequences (CDS) or exon regions of canonical protein-coding transcripts were excluded using BEDOPS[62] (v2.4.39).

To identify which of these genomic regions were under positive selection we used:

- OncodriveFML[38], as per Section 5.2.2, but with the "indel-max" argument used such that observed indels were treated as a set of substitutions with the corresponding functional impact being the maximum of said substitutions.
- ActiveDriverWGS[63] analyses if a region has higher mutational burden than expected, given mutational burden around local background sequence, applying Poisson generalised linear regression modelling.
- Negative binomial regression modelling method to investigate if there is a higher SNV burden than expected, given nucleotide context, which was also applied in Rheinbay *et al.*[64]. Regression modelling was adjusted by local mutational density and replication timing. Compared to ActiveDriverWGS, this method is not sensitive to potential neutral indel mutation hotspots.

To combine *P*-values from the three methods, which are not necessarily independent, we adopted a similar strategy as Gerstung, M. *et al.*[26] using Empirical Brown's method[65]. We adjusted for multiple-testing using the Benjamini-Hochberg procedure[66]. Post filtering of significant regions (i.e. Brown's *Q*-value < 0.001) was conducted to exclude those with accumulation of mutations caused by sequencing artefacts or mutational processes, by implementing the following inclusion criteria: (i) >2 mutations being present within the region; (ii) >50% of mutations located in a mappable genomic region (CRG alignabilibilty, DAC blacklisted, regions, and DUKE uniqueness) and (v) <50% of mutations resulting from APOBEC or AID signatures.

Empirical Brown's method is predicated on the assumption that *P*-values from each method approximately follow a scaled χ^2-distribution, which is however not necessarily the case with the output from the three algorithms. Acknowledging this, for reasons of pragmatism, we conservatively only discuss regions found to be significantly mutated by at least two of the three methods.

6. Somatic copy number alteration patterns

6.1. Copy number alteration classification

Copy number alterations (Section 2.5) were classified into six categories; homozygous deletion (HD), loss of heterozygosity (LOH), other loss of state (OLOS), no change, gain (GAIN) and big gain (AMP). The classification is also dependent on whether a genome was affected by whole genome duplication (Section 2.7) and if there existed intra-tumour heterogeneity in CNA states for a patient. If subclonal CNAs were detected, the copy

number state with the largest cell fraction was considered as the primary state. Classification details are found in Supplementary Table 25.

6.2. Enrichment of copy number alterations

6.2.1 Preparing GISTIC input and Initialization

Recurrent arm-level copy number events, as well as focal amplifications and deletions, were identified using Genomic Identification of Significant Targets in Cancer[67] (GISTIC, v2.0.2.3). Chromosomal coordinates and major (nMaj) and minor (nMin) copy number states were obtained for each copy number segment identified (Section 2.5). We selected the copy number (nMaj and nMin) of the dominant clone (*i.e*. the population with the largest tumour cell fraction) for analysis.

Tumours with and without WGD (ploidies assumed to be 4 and 2 respectively) were treated differently when calculating per-segment normalised copy numbers (SegCN). SegCN was thresholded to a minimum of -2 and maximum of 2.

For non-WGD tumours, per-segment normalised copy number was calculated as:

$$\text{SegCN} = (\text{nMaj} + \text{nMin}) - 2 \qquad (8)$$

For non-WGD tumours from males, X chromosome per-segment normalised copy number was calculated as:

$$\text{SegCN} = (\text{nMaj}+\text{nMin}) - 1 \qquad (9)$$

For WGD tumours, per-segment normalised copy number was calculated as:

$$\text{SegCN} = [(\text{nMaj}+\text{nMin}) - 4]/2 \qquad (10)$$

For WGD tumours from males, X chromosome per-segment normalised copy number was calculated as:

$$\text{SegCN} = (\text{nMaj}+\text{nMin}) - 2 \qquad (11)$$

GISTIC was run using the following parameters: *-conf 0.99 -broad 1 -qvt 0.25 -genegistic 1 -gcm extreme -brlen 0.5 -rx 0 -twoside 1 -scent median -armpeel 1 -arb 1 -refgene hg38.UCSC.add_miR.160920.refgene.mat*

6.2.2 Prioritising likely gene targets of focal amplifications and deletions

Recurrent loci targeted with focal amplifications and deletions were analysed for candidate target genes. We annotated using the following criteria:

1. Overlap with commonly found genes at focal amplifications and deletions reported in a previous pan-cancer study that used GISTIC. Comparisons were made both with the overall pan-cancer GISTIC analysis, as well as GISTIC analysis restricted to the given tumour type. Special attention was given to genes identified as candidates by Zack *et al.*[68].
2. Overlap with Cosmic Cancer Gene Census genes and whether their annotated role (oncogene [OG], tumour suppressor gene [TSG] or ambiguous) is consistent with the copy number change (OG with amplifications and TSG with deletions)[46].
3. Overlap with our identified list of driver genes and whether their likely role (OG, TSG or ambiguous) is consistent with the copy number change (OG with amplifications and TSG with deletions).

Using the above criteria, consensus driver genes were manually assigned to peaks. Comparisons were made with all potential gene synonyms made available via the HUGO gene nomenclature name committee (https://www.genenames.org/).

Alterations from the broad analysis with $Q<0.05$ were taken to indicate recurrent arm-level events. Copy number segments comprising greater than 50% of the total chromosome arm length were defined as arm-level events.

For focal events identified by GISTIC, the "wide region" was used to compare the potential extent of overlap with copy number segments. Segments were defined as overlapping focal events if either the segment interval comprised greater than 50% of the focal region, or vice versa, using pybedtools[69] and bedtools (v2.3.0)[70].

If an overlapping copy number segment was annotated as HD or LOH (as described above), tumours were deemed to have specific arm-level or focal deletions. Similarly, tumours were considered to have specific arm-level or focal amplifications if an overlapping copy number segment was annotated as GAIN or AMP. In the case of subclonal CNAs, nMaj and nMin values corresponding to the largest cell fractions were used.

7. Somatic structural variation patterns

7.1. Classification of simple and complex structural variants

Simple and complex SV classification was performed by first grouping rearrangements identified using the graph-based approach into footprints and clusters using ClusterSV[22]. Rearrangement footprints represent positionally associated rearrangement breakpoint sets and rearrangement clusters represent sets of mechanistically related rearrangements. We described rearrangement footprints using the string notation approach outlined by Li, *et al.*[22]. Rearrangement clusters were classified as simple or complex events if they comprised ≤2 or ≥3 individual rearrangements respectively. Simple events were further classified as deletions, tandem duplications, balanced inversions, balanced translocations, unbalanced translocations or unclassified simple SVs using their string notation. Complex events were classified as chromothripsis, chromoplexy or unclassified complex SVs as described below.

Complex SVs were classified as chromothripsis events on the basis of the following criteria[71,72]:

- Did not meet the criterion for chromoplexy events (see below).
- Contained ≥2 interleaved intra-chromosomal rearrangements. This liberal threshold (as compared with ≥6, commonly imposed) was used to minimise false negatives due to a lack of interleaved clusters that have been documented to be an issue in kidney RCC[71].
- Contained a contiguous series of ≥4 genome segments oscillating between 2 copy number states, or ≥5 genome segments oscillating between 3 copy number states.
- There was no evidence that the distribution of intra-chromosomal fragment-join orientations diverged from a multinomial distribution with equal probabilities for each of the four rearrangement orientation categories (deletion-like, duplication-like and head-to-head and tail-to-tail inversions; false discovery rate >0.2).

Complex SVs were classified as chromoplexy events if they met all the following criteria:

- Consisted of between 3 and 30 rearrangements.

- Contained a chain of rearrangements spanning ≥3 chromosomes[22]. SV chains were defined using a graph-based approach, in which nodes represent breakpoints and are connected by an edge if they fall within 1Mb of each other but are not involved in the same rearrangement.
- ≥50% of rearrangement footprints in the cluster represent balanced translocations, either with a deletion bridge between the break ends or no observed copy number change.

7.2. Simple structural variation hotspots

A permutation-based approach, as applied by Glodzik *et al.*[73], was used to identify hotspot regions for each type (Section 7.1) of simple SV. Complex SVs were not considered to identify hotspots.

7.2.1 Evaluating relationships between genomic features and SV rates

Negative binomial regression was used to test associations between select genomic features and numbers of SVs of each simple class, which is later applied to simulate SVs and identify SV hotspots. The following features[73] were included in the models:

- Average total copy number across the tumour.
- The presence of genes highly or lowly expressed in ccRCC, with genes given this annotation if their respective mean RNA-Seq by Expectation-Maximization (RSEM) values were in the top 25% and bottom 75% protein-coding genes respectively in TCGA samples.
- Guanine-Cytosine content given reference genome GRCh38.
- ALU repeats and other genomic repeats, obtained from the University of California Santa Cruz (UCSC) Genome Browser[74].
- Segmental duplications, obtained from the Segmental Duplication Database[75].
- Replication timing, from embryonic kidney cells (HEK293, Int57383924) obtained from ReplicationDomain[76].
- DNase peaks, with DNase-seq data obtained from female adult (47 years) kidney tissue from Encode[77] (GRCh38, ENCAN876VFO, ENCODE4 v3.0.0-alpha.2).
- H3K36me3 peaks, with ChIP-seq data obtained from male adult (50 years) kidney tissue from Encode[77,78] (GRCh38, ENCAN946KXL, ENCODE4 v1.7.0).
- H3K9me3 peaks, with ChIP-seq data obtained from male adult (50 years) kidney tissue from Encode[77,78] (GRCh38, ENCAN127GOZ, ENCODE4 v1.7.0).
- Fragile Sites, obtained from Bignell *et al.*[79].

7.2.2 Simulating SVs

As part of the pipeline to identify SV hotspots, the expected number of SVs per 1Mb bin was generated conditional on genomic features. The simulated SVs preserved the distribution of each SV class (*i.e.* number and length based on the distance between intra-chromosomal SV break ends) from the observed SVs. The simulation comprised of the following steps:

- The genome was divided into non-overlapping 1Mb bins, and the genomic features of each bin were summarized and normalized to a mean of 0 and standard deviation of 1.
- The number of break ends expected in each bin was estimated based on the coefficients from the negative binomial regression model.
- For each observed SV, an SV was simulated by first sampling a bin under probabilities proportional to the expected numbers of break ends in each bin. Each corresponding partner break end was then simulated by selecting the position either upstream or downstream (with equal probability) with the pairwise breakpoint distance being the same as the observed SV. It was resimulated if either break end fell within an uncallable region (*i.e.* a telomere or centromere).
- Steps 1-3 were repeated 1,000 times to generate a null distribution of expected SV numbers for each of the 1Mb bins.

7.2.3 Identifying SV hotspots

Piecewise constant fitting (PCF) was used to identify regions of the genome containing greater numbers of SV break ends than expected by chance. SV break ends were first sorted by position and the distance between successive break ends calculated. PCF was then applied to the $\log_{10}$ of these inter-mutational distances (IMD). SV hotspots were identified by first computing the observed (d_i^{obs}) and expected (d_i^{exp}) number of breakends per base pair for each PCF segment (i):

$$d_i^{obs} = \frac{a_i}{s_i} \quad (12)$$

$$d_i^{exp} = \frac{\sum_{j=1}^{n} b_j}{s^{bin}{}_n} \quad (13)$$

Where a_i is the number of break ends in the segment, s_i is the length of the segment in base pairs, n is the number of bins overlapping the segment, b_j is the expected number of SVs in bin j, and s^{bin} is the bin size (1Mb). A simple SV enrichment factor (β_i^{simple}) is then computed for each PCF segment as:

$$\beta_i^{simple} = \frac{d_i^{obs}}{d_i^{exp}} \quad (14)$$

The PCF algorithm requires parameters γ (that controls the smoothness of the segmentation) and k_{min} (the minimum number of mutations in a segment). A choice of $\gamma = 10$ and $k_{min} = 4$ was made. False discovery rates (FDRs) at each β^{simple} value were estimated by applying PCF to both the observed and simulated SV sets and dividing the mean number of segments with a β^{simple} value at least as great in the simulated SV sets by the number of segments with a β^{simple} value at least as great in the observed SV set. SV hotspots where no SVs were supported by CNAs were considered potential artefacts and removed. Overlapping SV hotspots were collapsed.

7.3. Classification of SV hotspots as fragile sites

SV hotspots at potential fragile sites likely occur for mechanistic rather than selective reasons and were therefore not considered further. SV hotspots were annotated as overlapping fragile sites if they met at least three of the following six criteria:

- Encompassed a late replicating region[80], defined by mean Repli-Seq values ≤0. Replication timing data from ccRCC embryonic kidney cells (HEK293) was used and obtained from ReplicationDomain[76].
- Had low gene-density[81], defined by a threshold of five genes per Mb.
- Overlapped a gene greater than 300kb in size, given that fragile sites have been shown to occur in regions containing genes at least 300kb in size[82].
- The overlapping gene of greatest size was the focus of the SV enrichment. A focused gene was defined if the ratio of SV break point densities in the largest gene compared with the intergenic regions flanking 1Mb upstream and downstream of said gene was <5.
- Overlapped reported fragile sites obtained from NCBI or literature curation[79], which were mapped from NCRI36 to GRCh38 co-ordinates using LiftOver[74].

- Overlapped reported fragile sites from an analysis of the PCAWG cohort[83], which were mapped from GRCh37 to GRCh38 co-ordinates using LiftOver[74].

8. Mutational Processes

8.1. Characterising single-base-substitution, doublet-base-substitution and indel signatures

De novo extraction of single-base-substitution (SBS), doublet-base-substitution (DBS) and insertion and deletion (ID) signatures, including decomposition to known COSMIC signatures[7] (v3.2), was performed using SigProfilerExtractor[84]. SNV mutations were formatted with respect to tri-nucleotide context and if they are on a transcribed or untranscribed side of a gene (SBS288). All signatures were extracted using random initialization, 500 NMF replicates, and between 10,000 and 1,000,000 NMF iterations. We assumed the presence of between 1 and 30 SBS and ID signatures, and 1 and 20 DBS signatures. Optimal solutions were manually chosen considering solution stability across NMF replicates, observed mutational profile reconstruction error, and consistency with previously reported renal cancer signature compendiums[84,85].

8.2. Predicting homologous recombination deficiency

Homologous recombination deficient (HRD) tumours were classified using HRDetect[86,87]. HRDetect predicted HRD using six genomic features, specifically SBS3 and SBS8 activities, rearrangement signatures RS3 and RS5 activities, proportion of deletions with microhomology and HRD index. SigProfiler did not identify SBS3 in any tumours and its activity was therefore considered 0 in all tumours. SBS8 activity was estimated using SigProfilerExtractor. RS3 and RS5 activities were estimated using HRDetect, based on the rearrangement signatures characterised by Nik-Zainal *et al.*, [87]. Whilst HRDetect was trained on breast cancers, it performs well when applied to other cancers[86].

9. Mutation Timing

9.1 Timing of copy number alterations and somatic mutations

The timing of SNVs and CNAs was determined using MutationTimeR[26]. MutationTimeR classifies SNVs into four categories: early clonal, late clonal, subclonal, unspecified clonal. The program achieves this by estimating the timing of copy number changes via the ratio of duplicated and unduplicated mutations within

regions of copy number gain. Mutations acquired before the gain will be present on both copies of the gained allele while mutations acquired after the gain will only be present on one allele. Copy number losses can only be timed if the losses are "copy number neutral", that is, one allele is lost while the other allele is simultaneously gained. SNVs are annotated as early clonal or late clonal if they occur before or after the copy number change respectively. Mutations occurring in regions of the genome that have not undergone a CNA are unable to be timed and are annotated as unspecified clonal. Subclonal mutations are determined by estimation of the clonal frequency from the VAF, purity, coverage and copy number.

The clonal state of mutations, as defined by MutationTimeR[26], are extracted for the previously identified driver mutations. The sample odds ratio was calculated for both early/late and clonal/subclonal driver mutations with associated *P*-values from Fisher's exact test.

9.2 Relative ordering of driver events

The estimate of the time at which a CNA occurred, as described above, was calculated in terms of the "mutational time". It is possible to determine the relative ordering of driver mutations and focal CNAs across the cohort while working with mutational time. A league model approach was taken as previously described in Gerstung *et al.*,[26]. For each pair of driver mutations and focal CNAs, hereby referred to as event A and event B, a multinomial distribution was built corresponding to the likelihood of the following: event A occurs before event B, event B occurs before event A, or the ordering is unknown. It is important to note that the timing of mutations by MutationTimeR is always relative to the patient specific CNA on the given chromosome and care must be taken when comparing the timing of mutations across chromosomes. As an example, if a patient has a gain on chromosome 1 at 0.8 "mutational time" and a gain on chromosome 2 at 0.3 "mutational time", then an early mutation on chromosome 2 is more likely to have occurred before an early mutation on chromosome 1. This behaviour is reflected when generating the distributions. For each pair of events the multinomial distribution is sampled and points are awarded to the events: 2 points for the event drawn occurring earlier, 0 points for the event drawn occurring later, and 1 point if unknown ordering is drawn. The event ordering is determined by the final league table after all events have been drawn against each other. The distribution of the ordering is generated by running the league model 1000 times. In addition, the cohort was restricted to 75% of the sample size at random to account for anomalous samples. The entire procedure is performed on 1000 randomly selected subsets of the cohort and the final ordering distribution is aggregated across all results.

The true chronological time at which CNAs occurred can be estimated by taking into account numerous assumptions: the mutation rate is constant across a patient's lifetime, the mutation rate is constant across the whole genome, and the mutation rate is the same in both normal and tumour cells. Uncertainty in the modelling was estimated by varying the rate at which mutations accumulate in tumour cells, thereby simulating a shorter period of tumourigenesis.

10. Immune Profiling

10.1. Human Leukocyte Antigen (HLA) Typing

All genomes were HLA-typed using POLYmorphic loci reSOLVER[88] (POLYSOLVER). This outputs the six HLA-alleles across the three HLA-Class I genes of HLA-A, HLA-B and HLA-C for each patient.

10.2 Neoantigen Prediction

Neoantigens were predicted using the genome-guided *in silico* approach, personalized Variant Antigens by Cancer Sequencing[89] (pVAC-Seq), which applies eight methods (NetMHC, NetMHCpan, MHCflurry, SMM, NetMHCcons, SMMPMBEC, MHCnuggetsI, PickPocket) to predict the binding affinities of epitopes. Neoantigen binding strengths were defined as the mean strength from the eight methods.

Firstly, pVAC-Seq was applied to independently predict, per patient, epitopes and their binding affinity scores that arise as a result of non-synonymous mutations, based on the HLA-alleles typed by POLYSOLVER. Subsequently, pVAC-Seq derives the transcribed wild-type and mutant-type (8-10-mer) peptides corresponding to each non-synonymous mutation. Each of the peptides were scored based on binding affinity (nM) with respect to the patient’s major histocompatibility complex class I (MHC-I) molecule. A given peptide is classified as a neoantigen if the peptide meets the following conditions:

- Has a binding affinity ≤500nM.
- Corresponds to a canonical transcript.
- Is novel with respect to the human proteome.

When defining neoantigens, all patient-specific alleles were used and immune escape mechanisms were disregarded.

10.3 Immune Escape

We investigated three immune escape mechanisms, specifically: (i) a non-synonymous mutation in any of the three (HLA-A,-B,-C) HLA Class I genes; (ii) loss of heterozygosity (LOH) in any of the three HLA-I genes or (iii) any inactivating mutation in a list of 22 antigen presenting genes. We labelled a tumour sample with positive immune escape status if they exhibited any one of (i)-(iii).

HLA gene mutations were found using POLYSOLVER. This uses a combination of MuTect to check for nonsynonymous SNVs and Strelka for insertions and deletions in HLA-aligned reads. HLA LOH was predicted using Loss of Heterozygosity in Human Leukocyte Antigen[90] (LOHHLA). The HLA typing from POLYSOLVER was parsed into LOHHLA, in addition to the full tumour and germline bam files, per patient, as input. LOHHLA crops the two BAM files to the hg19 HLA-region on chromosome 6 by default. Instead, this was edited to conform to the hg38 HLA-region chr6:28510120-33480577. LOHHLA was run with the mapping and fishing steps turned on. The number of mismatch sites between any two allele pairs was set to >10 and the minimum coverage filter at these sites was set to 10. LOHHLA did not make predictions for genes of patients that did not meet one or both of these thresholds.

To define HLA-LOH we used the same definition as Cornish *et al.*[91]

- Presence of allelic imbalance, with the difference in evidence of the two alleles fulfilling $P < 0.01$.
- The copy number of the lost allele was <0.5 with a confidence interval <0.7.
- The copy number of the kept allele was >0.7.
- The number of mismatched sites between alleles was >10.

We curated a set of 22 antigen presenting genes (APGs) spanning the genetic components of antigen presenting machinery, the IFN-γ pathway, the PF-L1 receptor, the CD58 receptor, and epigenetic escape via *SETDB1* (*APLNR, B2M, CANX, CALR, CD274, CD58, CIITA, ERAP1, ERAP2, IRF2, IFNGR1, IFGNR2, JAK1, JAK2, NLRC5, PDIA3, RFX5, SETDB1, STAT1, TAPBP, TAP1, TAP2*)[92,93]. Any of the 22 APGs were labelled as inactivated if the gene met any one of the following three conditions:

- Monoalleic or bialleic clonal loss-of-function mutation annotated with any of the VEP calculated consequences: 'frameshift variant', 'stop gained', 'stop lost', 'splice acceptor variant', 'splice donor variant', 'splice region variant' or 'start lost'.
- Biallelic clonal non-synonymous mutation, or a monoalleic clonal non-synonymous mutation plus loss of heterozygosity, annotated with any of the following VEP calculated consequences: 'transcript ablation', 'transcript amplification', 'inframe insertion', 'inframe deletion', 'missense variant' or 'protein altering variant'.

- Homozygous deletion.

11. Correlating Clinicopathological and Mutational Variables

11.1. Correlations with Mutational Attributes

The correlation between the clinical features and molecular variables were assessed by chi-squared tests with a two-sided *P*-value <0.05 being considered statistically significant. For associations between genetic features, negative binomial regression was applied for HLA allele and neoantigens count, and otherwise logistic regression was used. Some associations are not reported due to convergence issues or a lack of events in binary variables. To adjust for confounding, regression models were adjusted by patient sex, age of sampling, tumour stage and tumour grade (Supplementary Table 1). In considering the relationship between mutational signatures and patient survival were stratified into high and low activity (*i.e*. above or below the median activity of each signature).

11.2. Survival Analysis

Overall survival (OS) and cancer specific survival (CSS) was defined as the time from the date of sampling to death from any cause. Individuals were excluded if tumour sampling occurred prior to 1 January 2015 or the time between ccRCC diagnosis and tumour sampling was more than one year. Kaplan–Meier survival curves were generated and the homogeneity between groups was evaluated with the log-rank test. Progression-free survival (PFS) was defined as the time from the date of sampling to radiological progression. Cox regression analysis was used to estimate hazard ratios (HRs) and respective 95% confidence intervals (CI), and adjustment for clinical variables (see Section 11.1) was performed by multivariable analysis (Supplementary Table 1). In assessing the survival relationship with driver gene mutations or CNAs we only evaluated those with a carrier frequency >5%.

SUPPLEMENTARY FIGURES

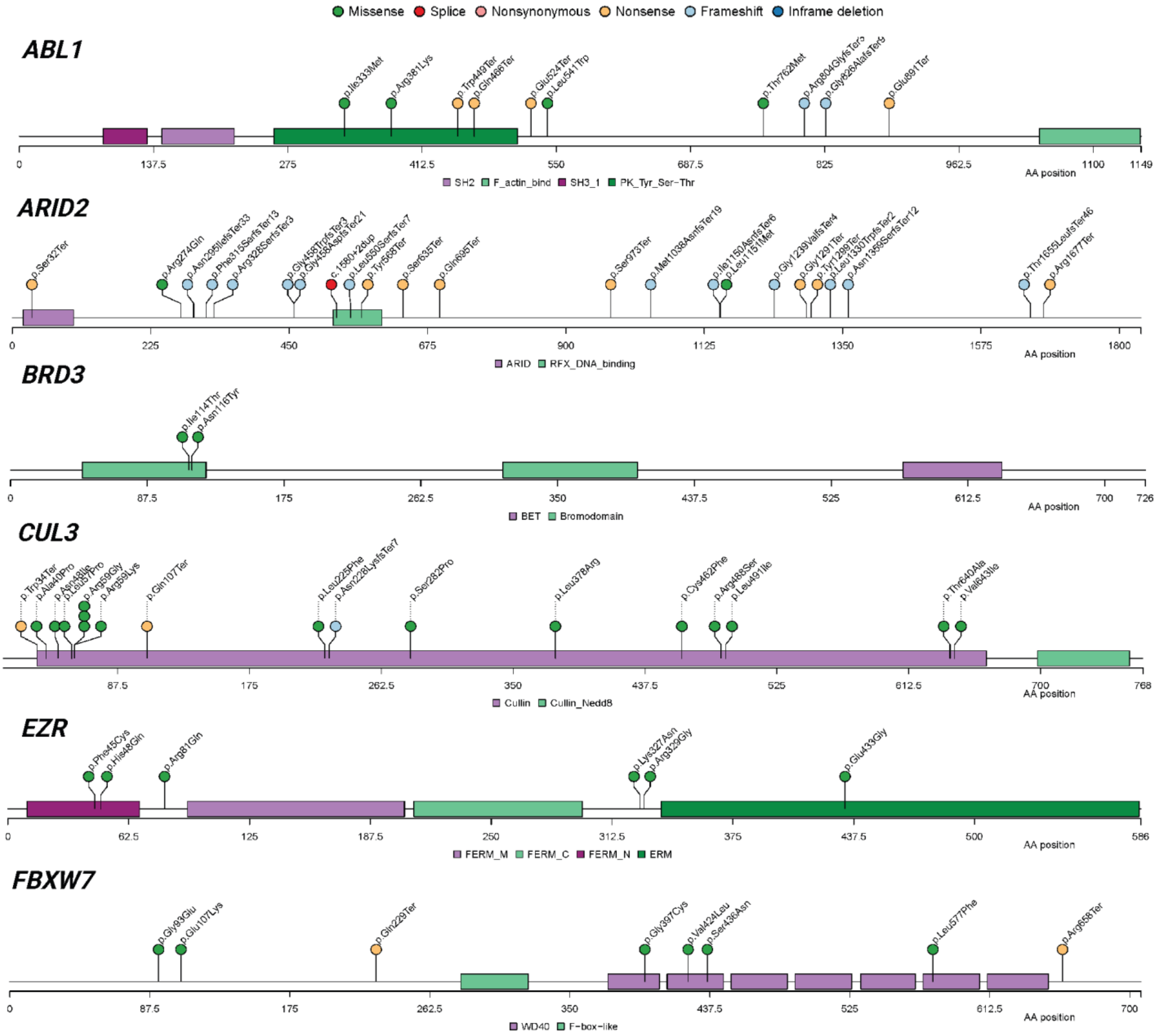

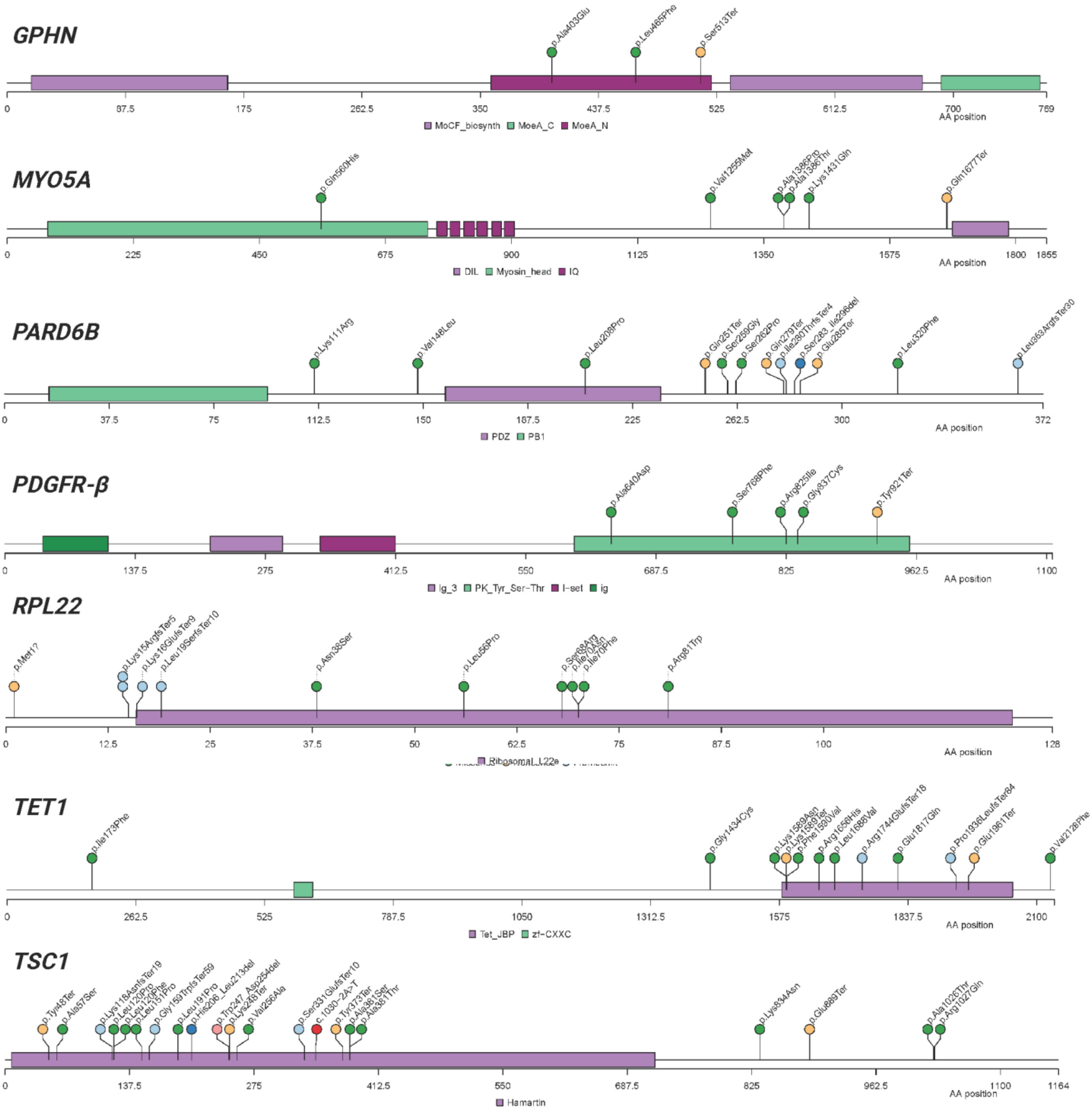

Supplementary Figure 1: Mutational distribution plots for the 13 novel genes. (a) *ABL1*; **(b)** *ARID2*; **(c)** *BRD3*; **(d)** *CUL3*; **(e)** *EZR*; **(f)** *FBXW7*; **(g)** *GPHN*; **(h)** *MY05A*; **(i)** *PARD6B*; **(j)** *PDGFR-β*; **(k)** *RPL22*; **(l)** *TET1*; **(m)** *TSC1*. The colours of the circles represent the predicted consequence of each mutation. The domains are annotated using Pfam.

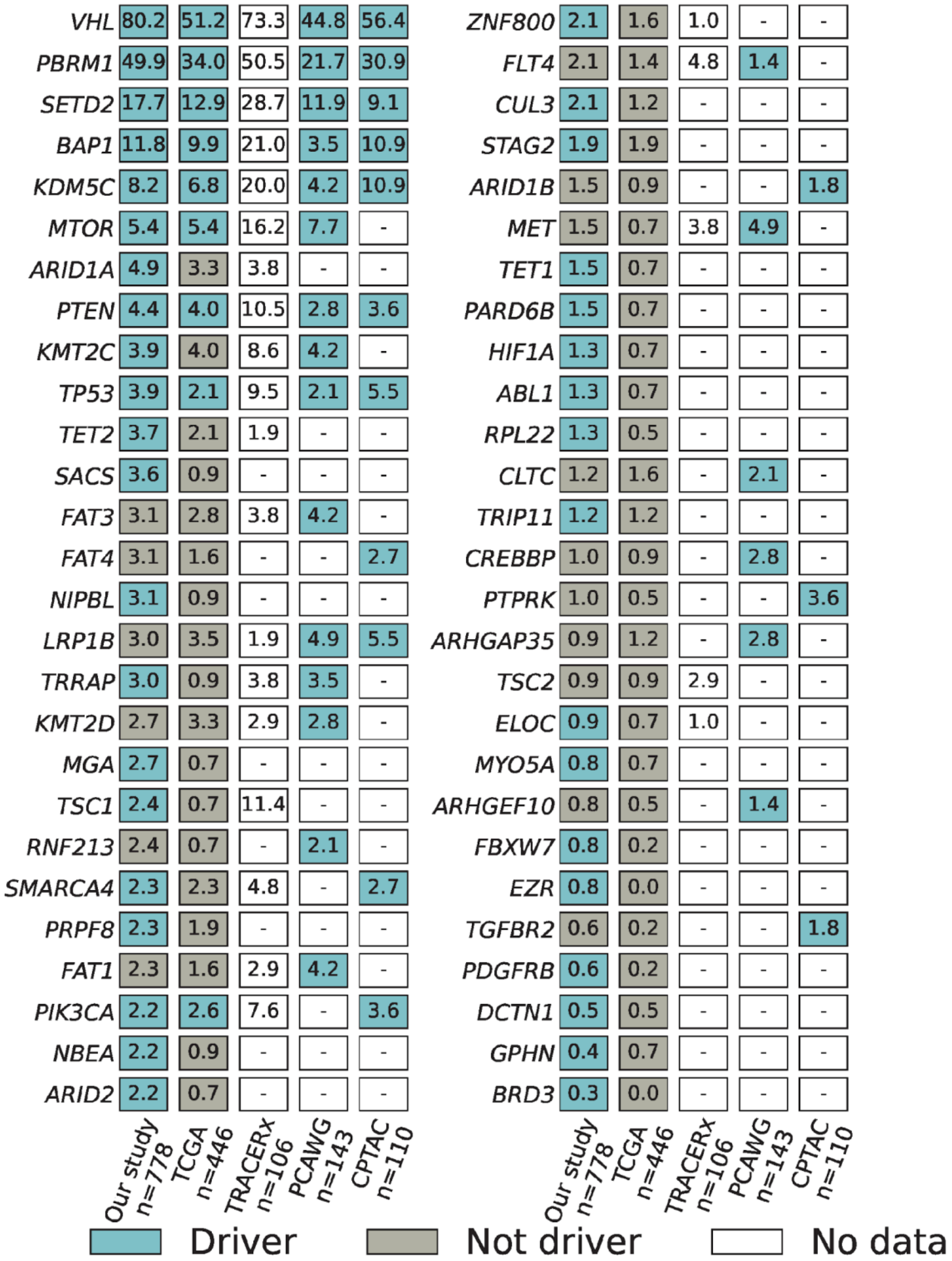

Supplementary Figure 2: Percentage of tumour samples with non-synonymous mutations in driver genes for our study, TCGA, TRACERx, PCAWG and CPTAC. Genes in blue refer to those designated as driver genes in respective studies. TCGA data based on whole exome sequencing of 446 tumours[49]. PCAWG data based on whole genome

sequencing of 143 tumours[29]. CPTAC data based on whole exome sequencing of 110 tumours[51]. TRACERx data based on targeted panel sequencing of 106 tumours with clonal and subclonal mutations being reported[50].

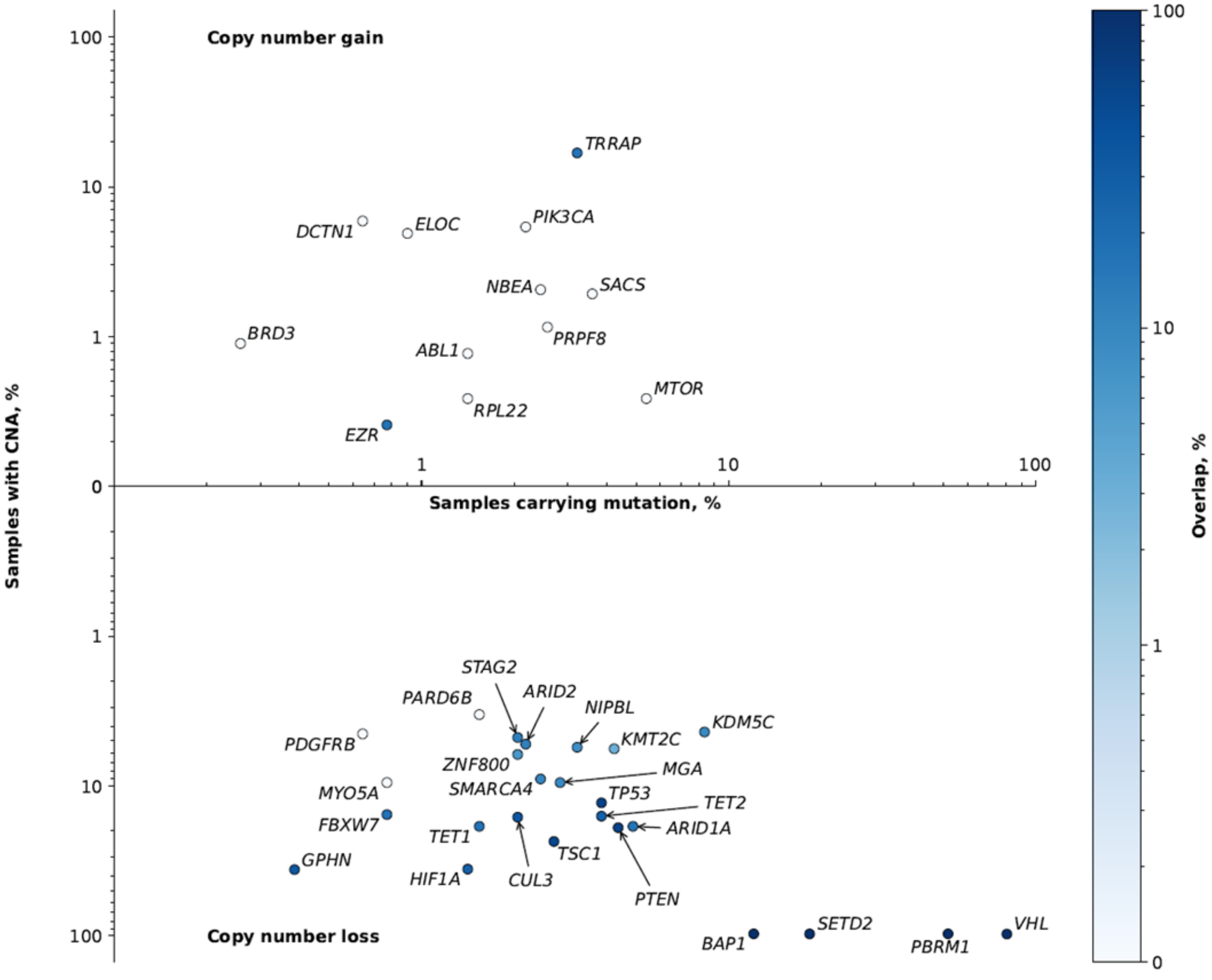

Supplementary Figure 3: **Percentage of tumour samples with non-synonymous mutations and copy number alterations in driver genes.** The colour scale corresponds to the degree of overlap, that is, the percentage of samples with a non-synonymous mutation that also have a copy number alteration that affects the given gene.

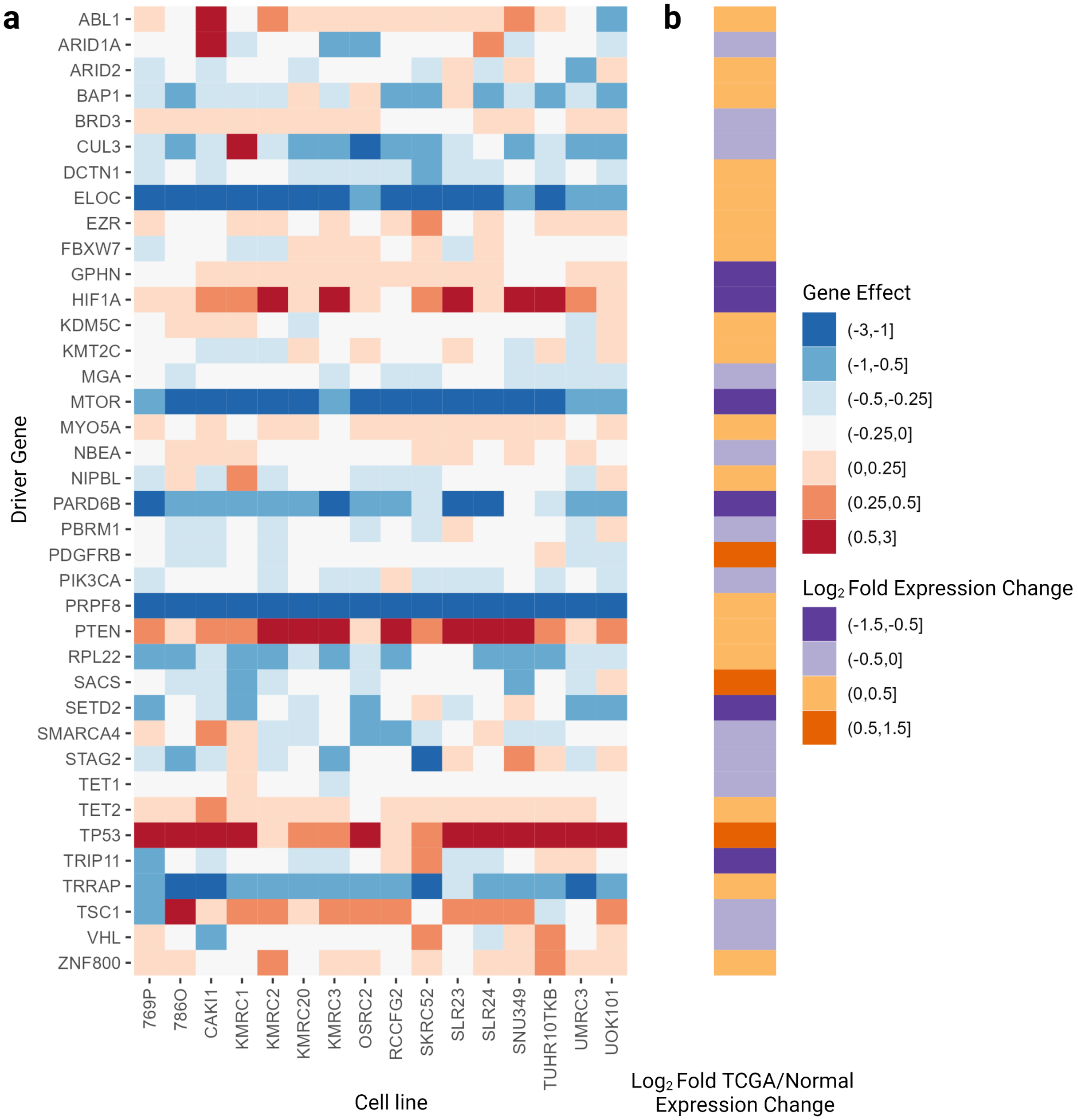

Supplementary Figure 4: (a) Driver effect on cell fitness given CRISPR loss-of-function screens from the DepMap consortium[52,53]; **(b)** Log_2 fold difference in the median expression between tumour and normal kidney tissue based on combined TCGA and GTEx data[54,55].

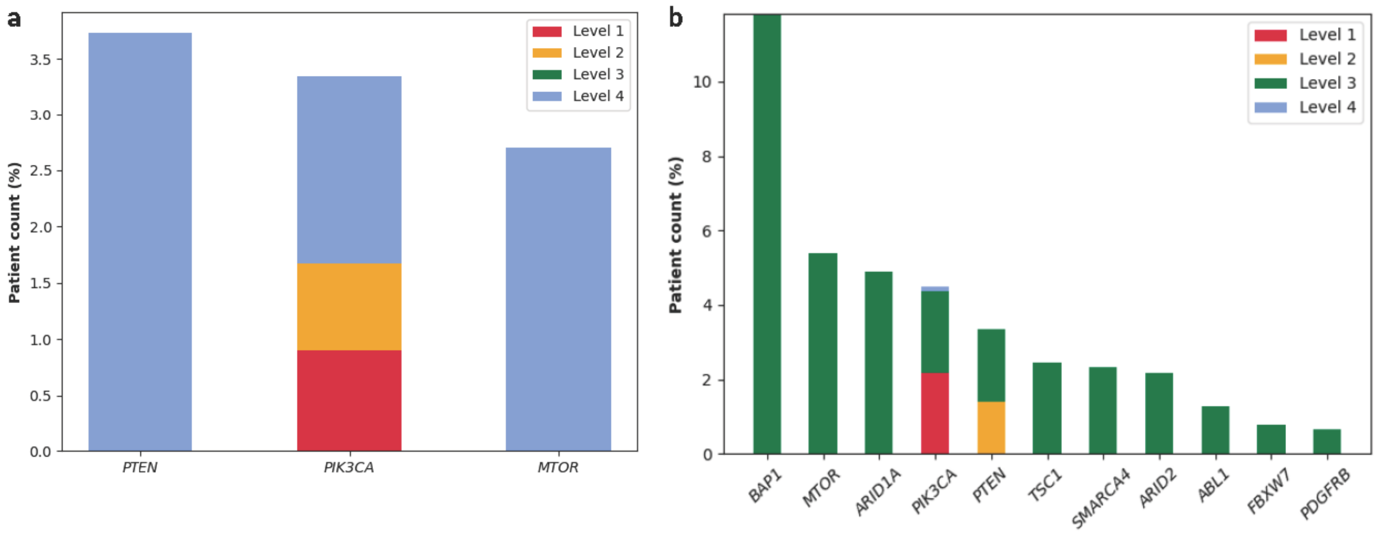

Supplementary Figure 5: Actionability of driver mutations. Number of patients benefiting from at least one treatment given a specific mutation or altered gene based on: **(a) OncoKB Knowledge Base**[58]**.** Level 1 - FDA-recognised biomarker predictive of response to an FDA-approved drug in this condition; Level 2 - Standard care biomarker recommended by the NCCN or other professional guidelines predictive of response to an FDA-approved drug in this indication; Level 3 - Compelling clinical evidence supporting the biomarker as being predictive of response to a drug in this indication/Standard care or investigational biomarker predictive of response to an FDA-approved or investigational drug in another indication; Level 4 - Compelling biological evidence supporting the biomarker as predictive; **(b) COSMIC Mutation Actionability in Precision Oncology**[7]**.** Level 1 - Approved marketed drug with demonstrated efficacy at the mutation; Level 2 - Phase 2/3 clinical results meeting primary outcome measures; Level 3 - Drug in ongoing clinical trials; Level 4 - Case studies.

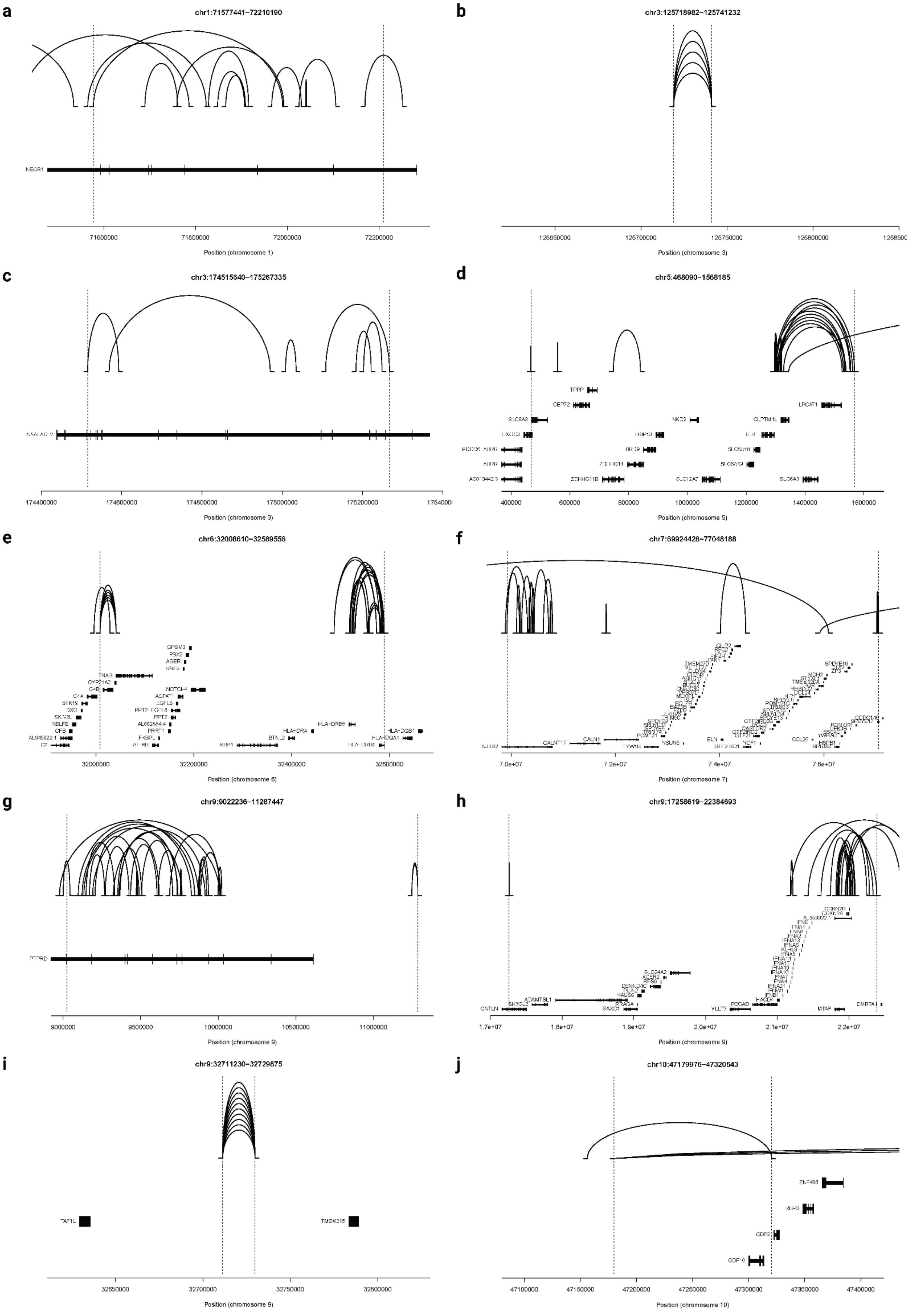
a
chr1:71577441–72210190
NEGR1
Position (chromosome 1)
b
chr3:125718982–125741232
Position (chromosome 3)
c
chr3:174515640–175267335
Position (chromosome 3)
d
chr5:468090–1568185
Position (chromosome 5)
e
chr6:32008610–32589556
Position (chromosome 6)
f
chr7:69924428–77048188
Position (chromosome 7)
g
chr9:9022236–11287447
PTPRD
Position (chromosome 9)
h
chr9:17258619–22384693
Position (chromosome 9)
i
chr9:32711230–32729875
TAF1L
TMEM215
Position (chromosome 9)
j
chr10:47179976–47320543
ZNF488
GDF2
GDF10
Position (chromosome 10)

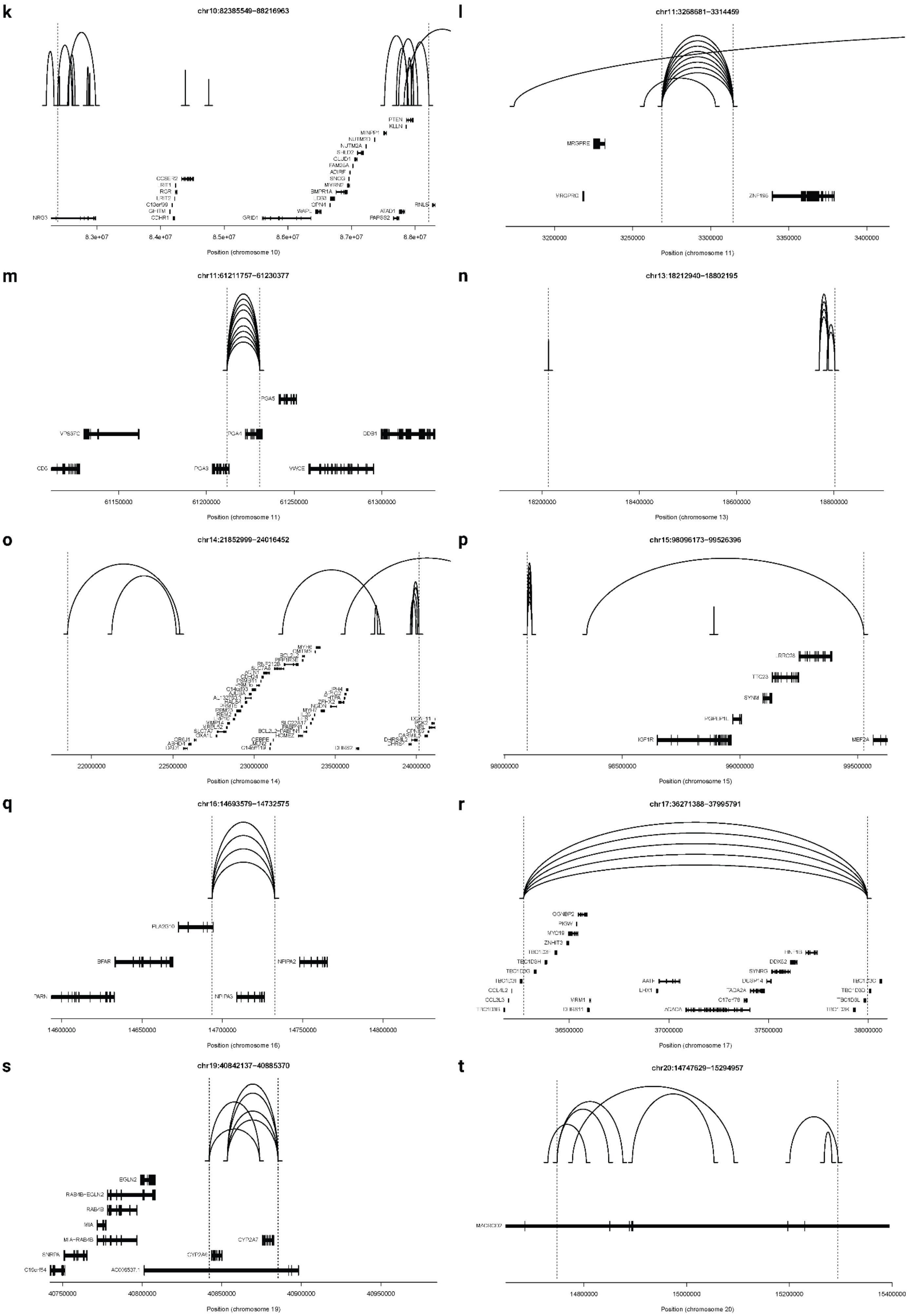
k
chr10:82385549-88216963
Position (chromosome 10)
PTEN
KLLN
MINPP1
BMPR1A
LDB3
OPN4
WAPL
GRID1
ATAD1
PAPSS2
RNLS
NRG3
CDHR1
l
chr11:3268681-3314459
Position (chromosome 11)
MRGPRE
MRGPRG
ZNF195
m
chr11:61211757-61230377
Position (chromosome 11)
PGA5
VPS37C
PGA4
DDB1
CD5
PGA3
VWCE
n
chr13:18212940-18802195
Position (chromosome 13)
o
chr14:21852999-24016452
Position (chromosome 14)
p
chr15:98096173-99526396
Position (chromosome 15)
LRRC28
TTC23
SYNM
PGPEP1L
IGF1R
MEF2A
q
chr16:14693579-14732575
Position (chromosome 16)
PLA2G10
BFAR
NPIPA2
PARN
NPIPA3
r
chr17:36271388-37995791
Position (chromosome 17)
GGNBP2
PIGW
MYO19
ZNHIT3
HNF1B
DDX52
SYNRG
DUSP14
LHX1
TADA2A
AATF
ACACA
s
chr19:40842137-40885370
Position (chromosome 19)
EGLN2
RAB4B-EGLN2
RAB4B
MIA
MIA-RAB4B
CYP2A7
SNRPA
CYP2A6
C19orf54
t
chr20:14747629-15294957
Position (chromosome 20)
MACROD2

Supplementary Figure 6: Breakpoint plots of deletion (structural variant) hotspot regions. (a) chr1:71577441-72210190; **(b)** chr3:125718982-125741232; **(c)** chr3:174515640-175267335; **(d)** chr5:468090-1568185; **(e)** chr6:32008610-32589556; **(f)** chr7:69924428-77048188; **(g)** chr9:9022236-11287447; **(h)** chr9:17258619-22384693; **(i)** chr9:32711230-32729875; **(j)** chr10:47179976-47320543; **(k)** chr10:82385549-88216963; **(l)** chr11:326861-3315549; **(m)** chr11:61211757-61230377; **(n)** chr13: 18212940-18802195; **(o)** chr14:21852999-24016452; **(p)** chr15:98096173-99526396; **(q)** chr16:14693579-14732575; **(r)** chr17:36271388-37995791; **(s)** chr19:40842137-40885370; **(t)** chr20:14747629-15294957.

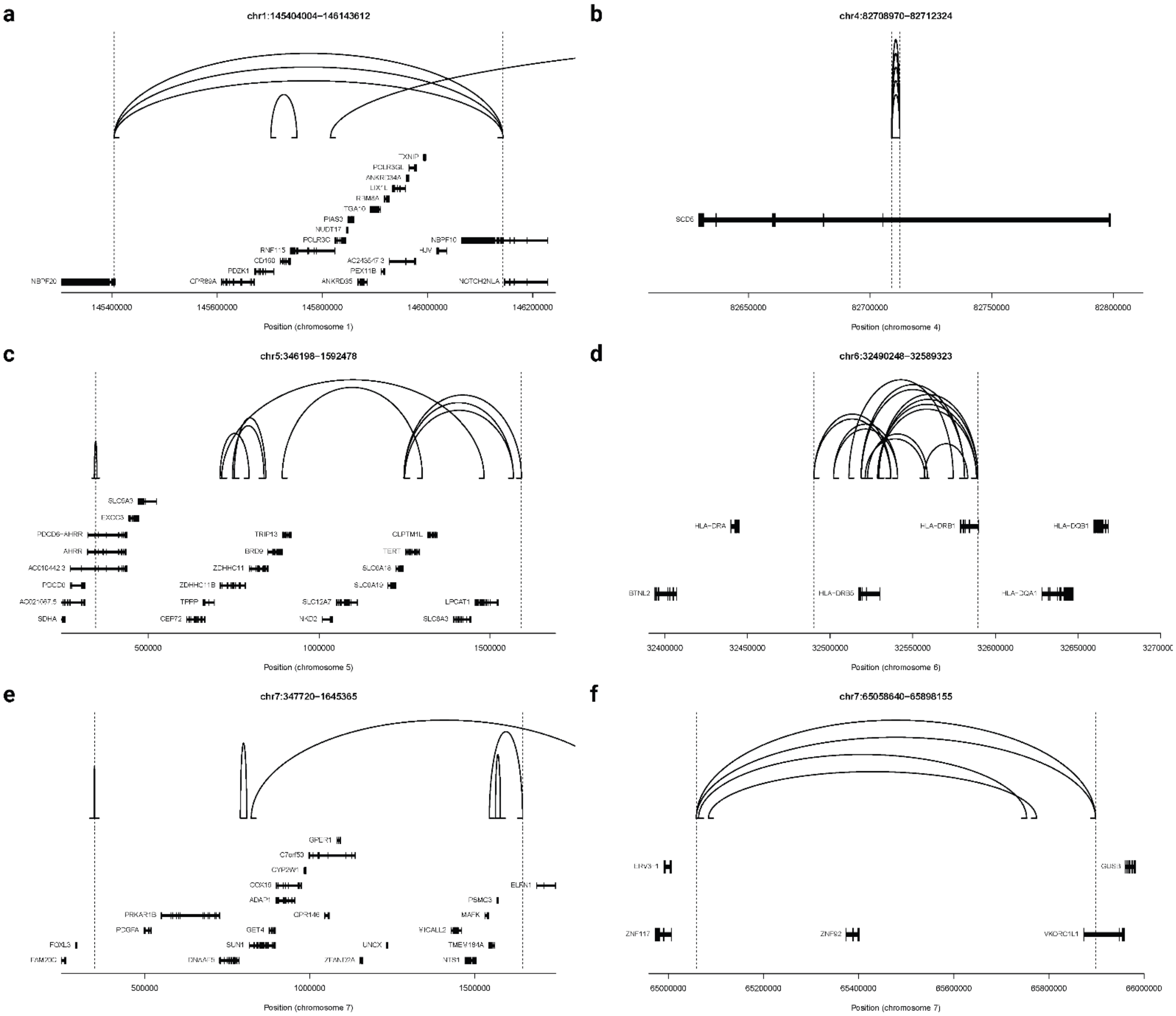
a
chr1:145404004–146143612
Position (chromosome 1)
b
chr4:82708970–82712324
Position (chromosome 4)
c
chr5:346198–1592478
Position (chromosome 5)
d
chr6:32490248–32589323
Position (chromosome 6)
e
chr7:347720–1645365
Position (chromosome 7)
f
chr7:65058640–65898155
Position (chromosome 7)

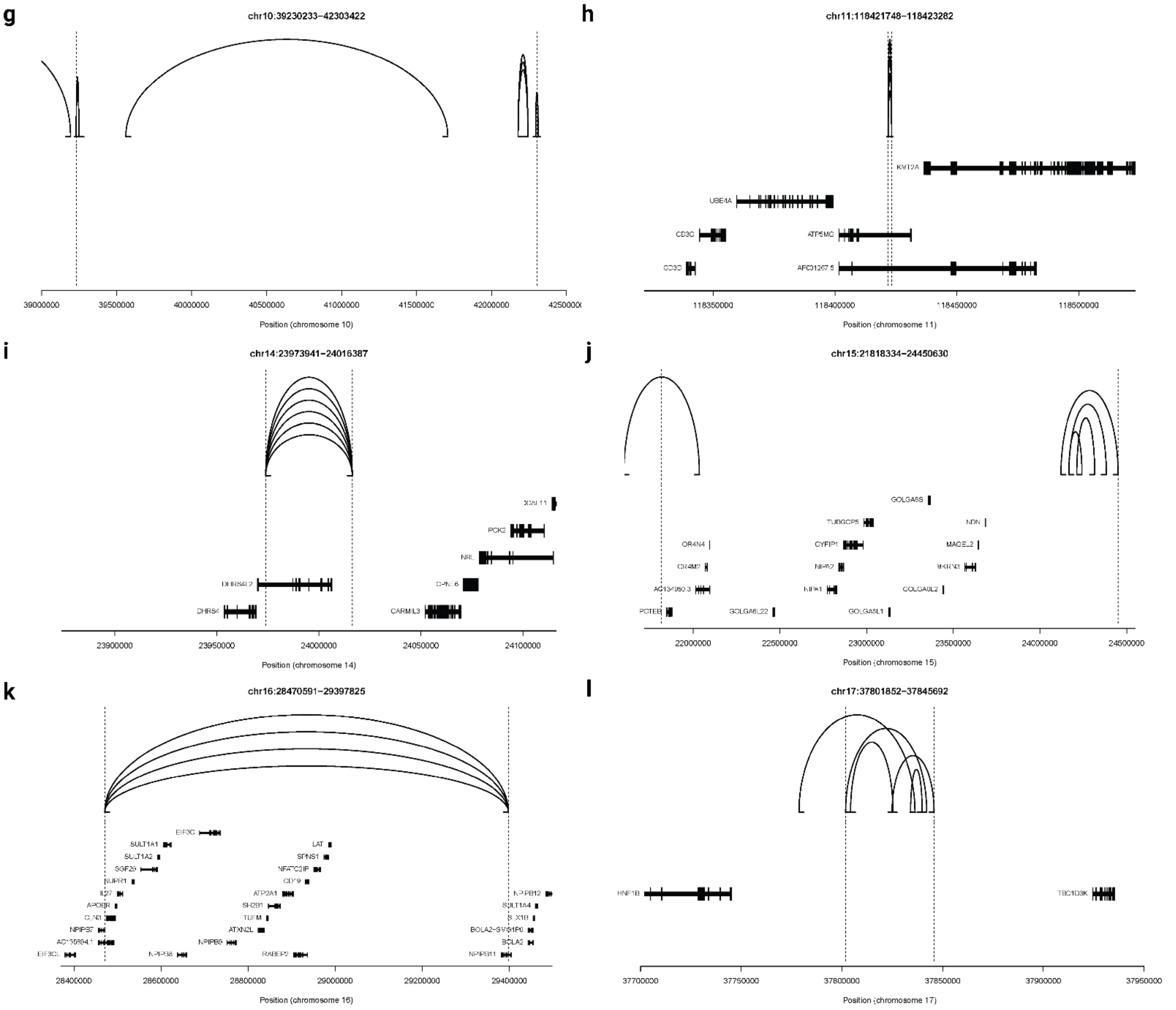

Supplementary Figure 7: Breakpoint plots of tandem duplication (structural variant) hotspot regions. (a) chr1:145404004-146143612; **(b)** chr4:82708970-82712324; **(c)** chr5:346198-1592478; **(d)** chr6:32490248-32589323; **(e)** chr7:347720-1645365; **(f)** chr7:65058640-65898155; **(g)** chr10:39230233-42303422; **(h)** chr11:118421748-118423292; **(i)** chr14:23973941-24016387; **(j)** chr15:21818334-24450630; **(k)** chr16:28470591-29397825; **(l)** chr17:37801852-37845692.

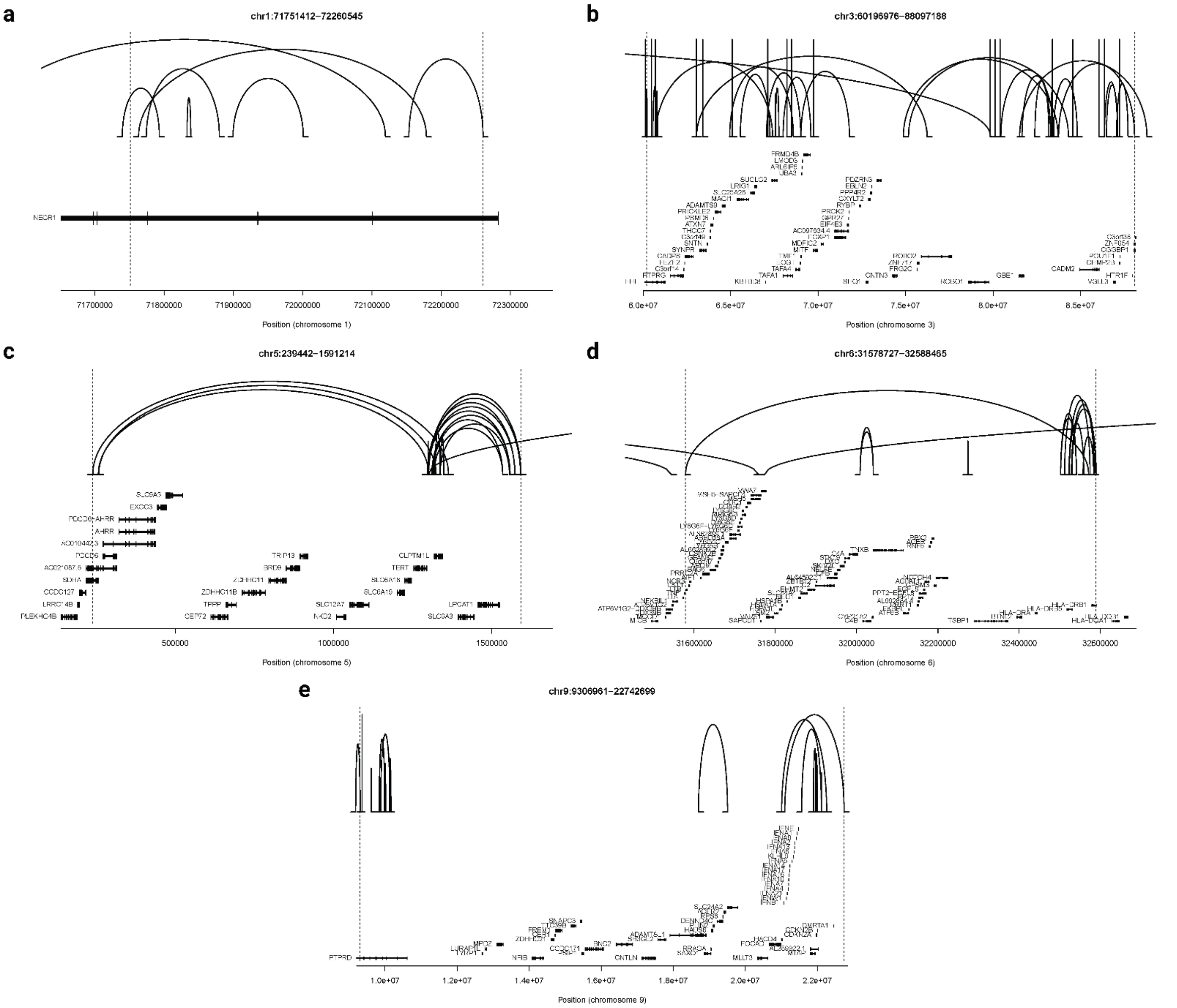

Supplementary Figure 8: Breakpoint plots of simple unclassified structural variant hotspot regions. (a) chr1:71751412-72260545; **(b)** chr3: 60196976-88097188; **(c)** chr5: 239442-1591214; **(d)** chr6:31578727-82588465; **(e)** chr9: 9306961-22742699.

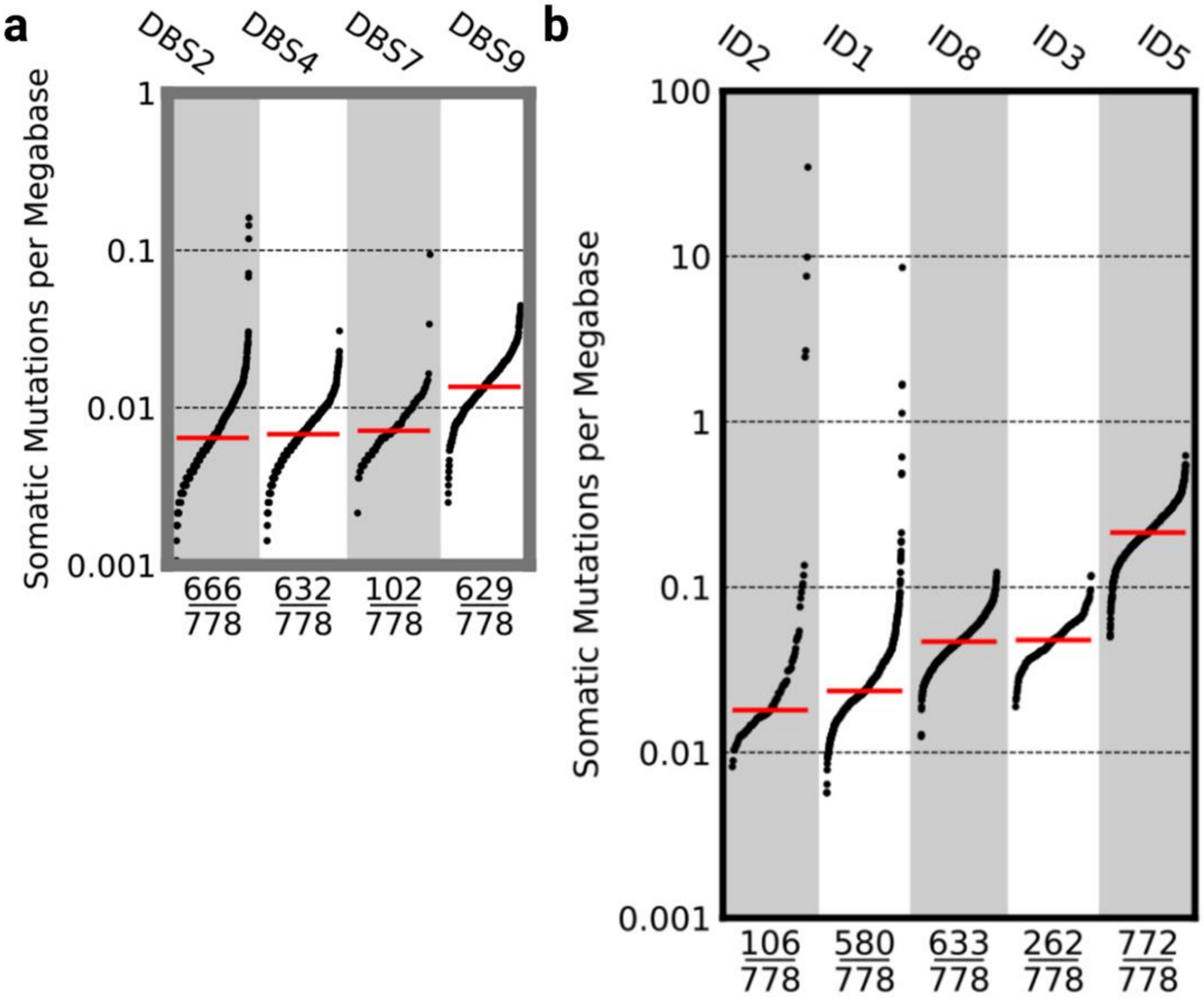

Supplementary Figure 9: The mutational burden of tumour samples and mutational signatures. (a) Doublet-base substitution COSMIC signatures (*n*=778); **(b)** Indel COSMIC signatures (*n*=778).

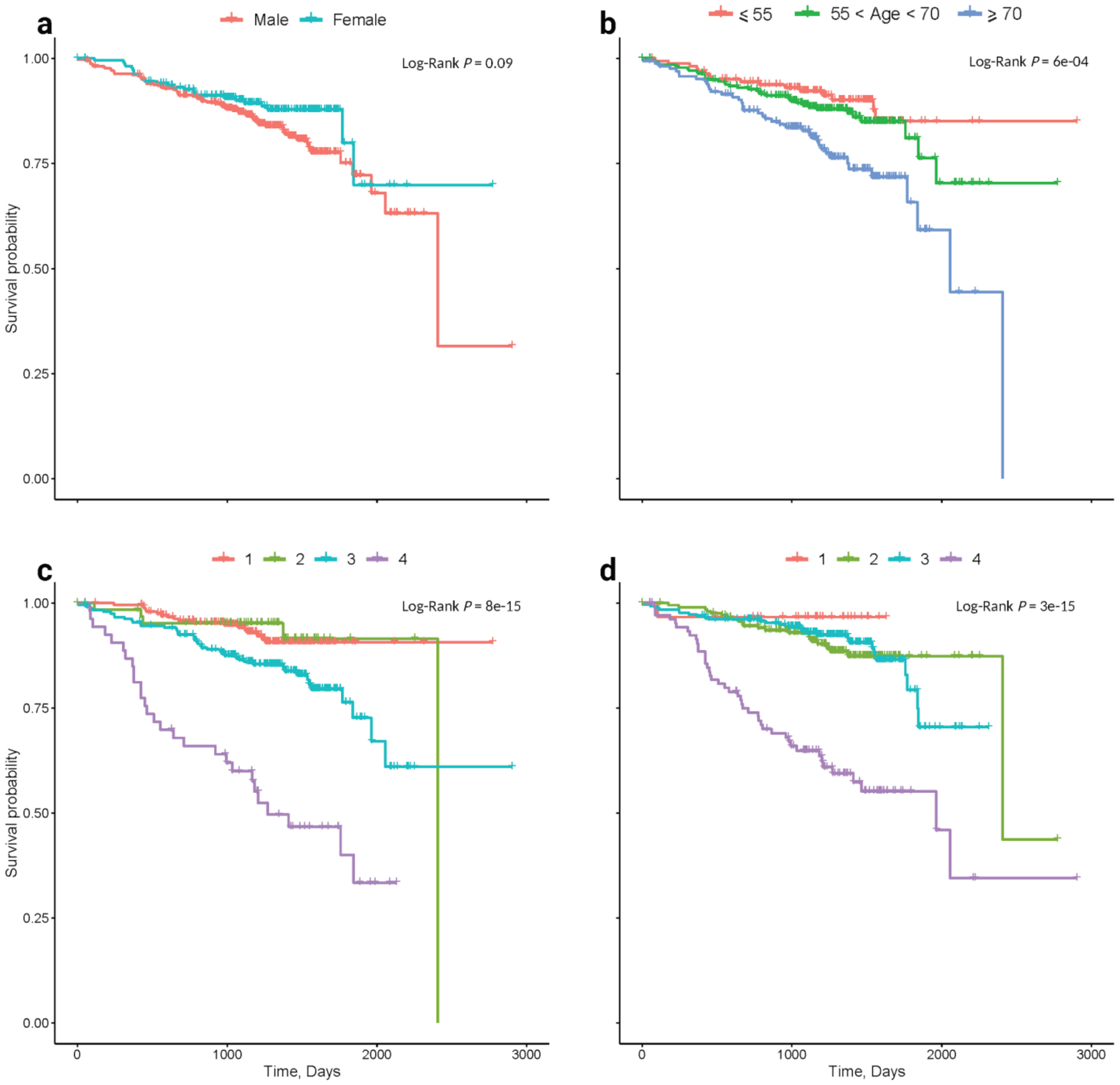

Supplementary Figure 10: Kaplan Meier curves of overall survival. Relationship with **(a)** sex; **(b)** age; **(c)** tumour stage; **(d)** tumour grade. Log-Rank *P* refers to the Log-Rank test.

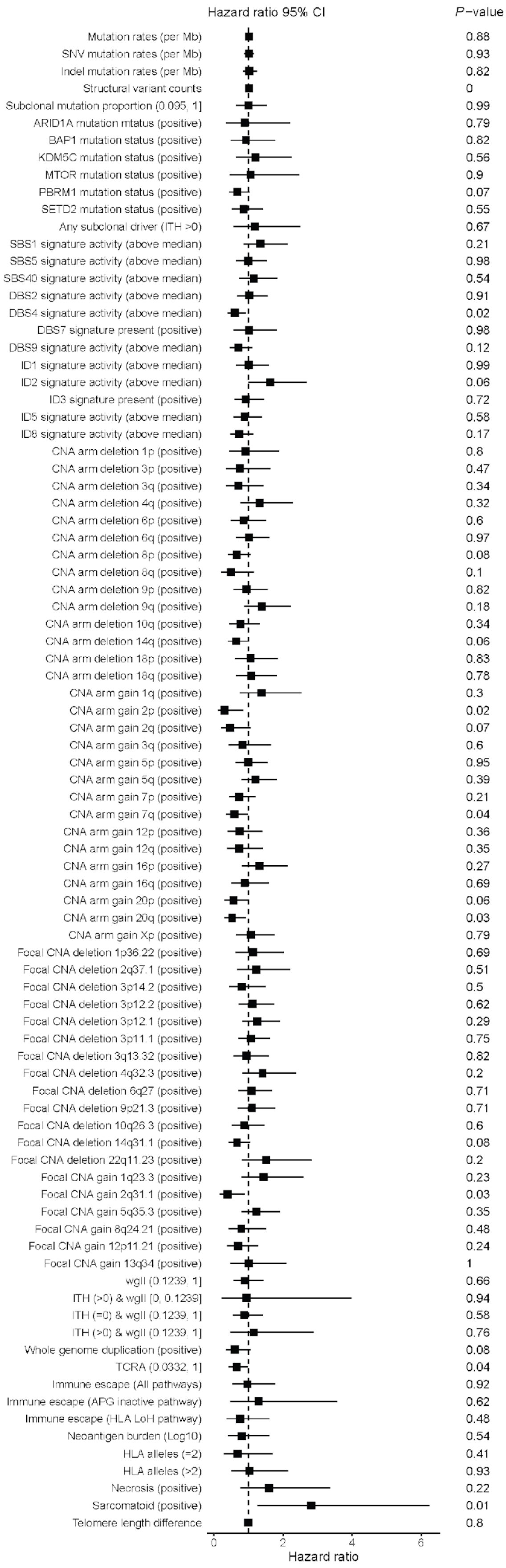

Supplementary Figure 11: Cox proportional hazards regression estimates for overall survival. The hazard ratios are reported with their 95% confidence intervals and corresponding *P*-values.

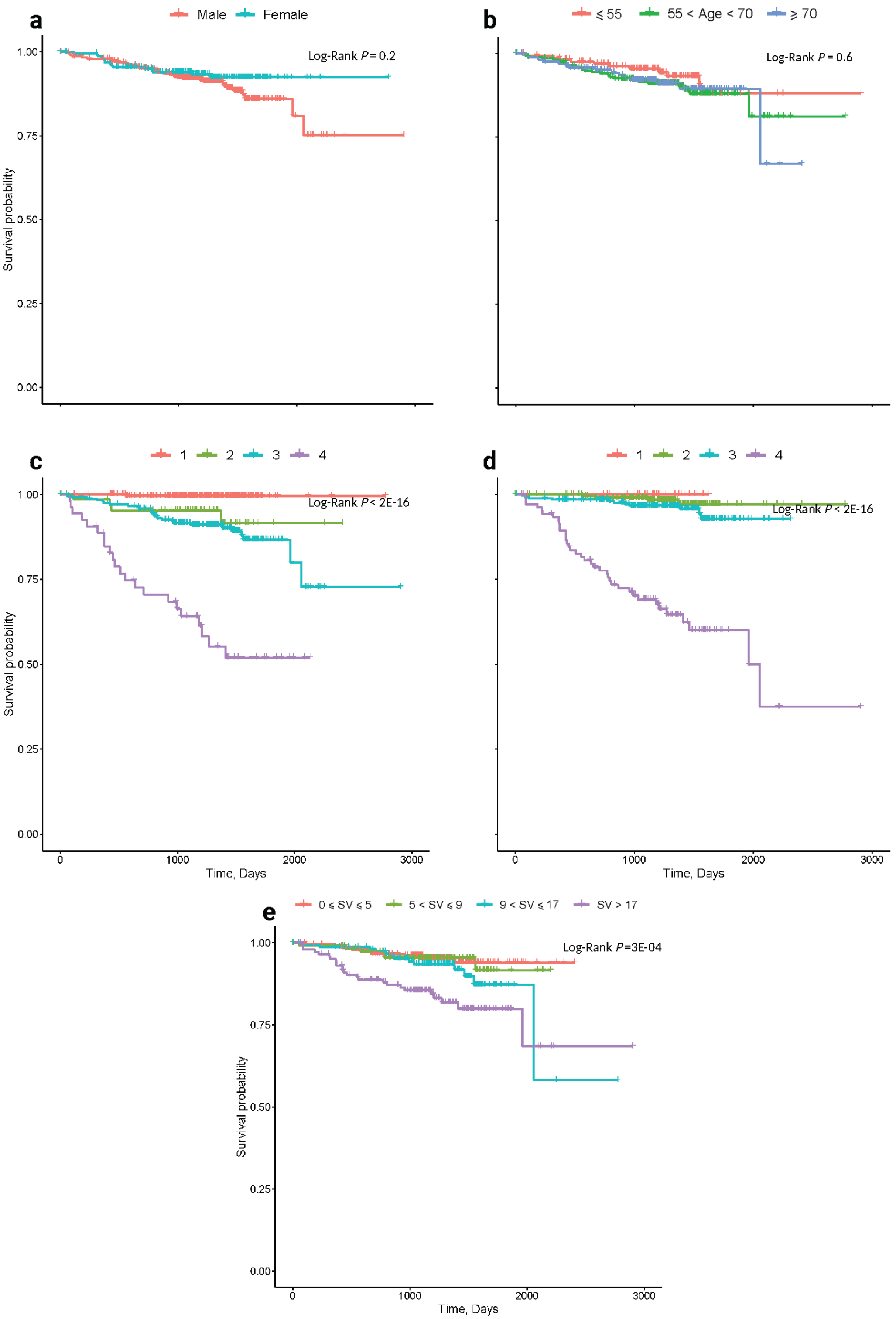

Supplementary Figure 12: Kaplan Meier curves of cancer specific survival. **(a)** sex; **(b)** age; **(c)** tumour stage; **(d)** tumour grade; **(e)** *VHL* Mutation Status. Log-Rank *P* refers to the Log-Rank test.

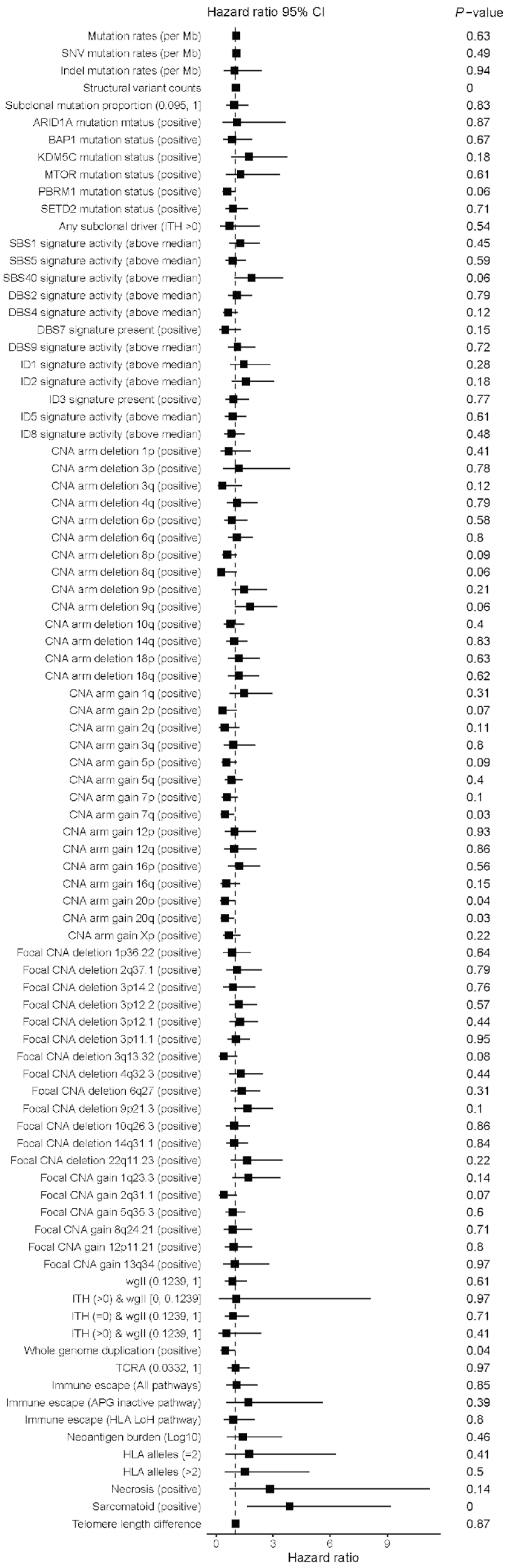

Supplementary Figure 13: Cox proportional hazards regression estimates for cancer specific survival. The hazard ratios are reported with their 95% confidence intervals and corresponding *P*-values.

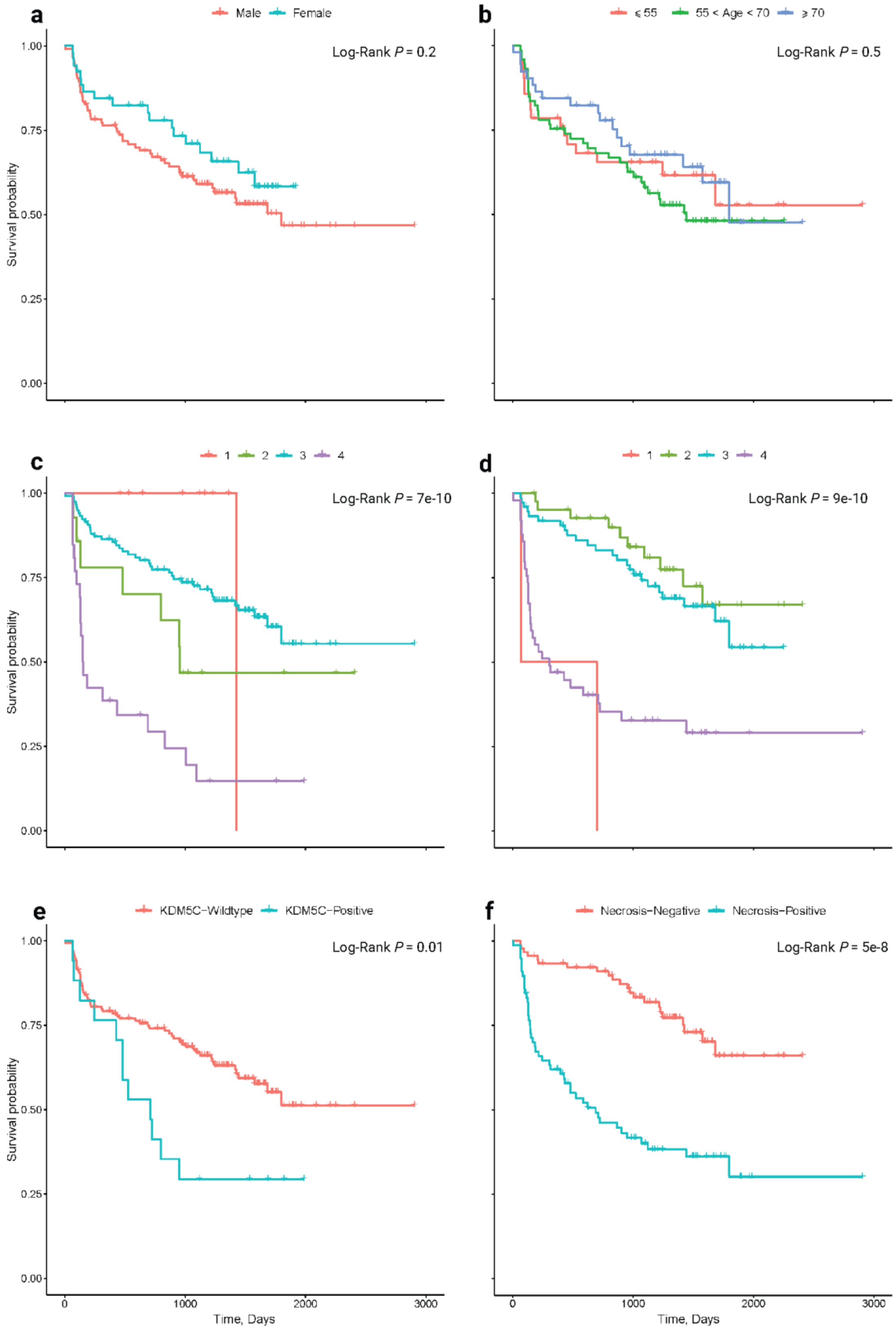

Supplementary Figure 14: Kaplan Meier curves of progression free survival. (a) sex; **(b)** age; **(c)** tumour stage; **(d)** tumour grade; **(e)** *KDM5C* mutation status; **(f)** necrosis. Log-Rank *P* refers to the Log-Rank test.

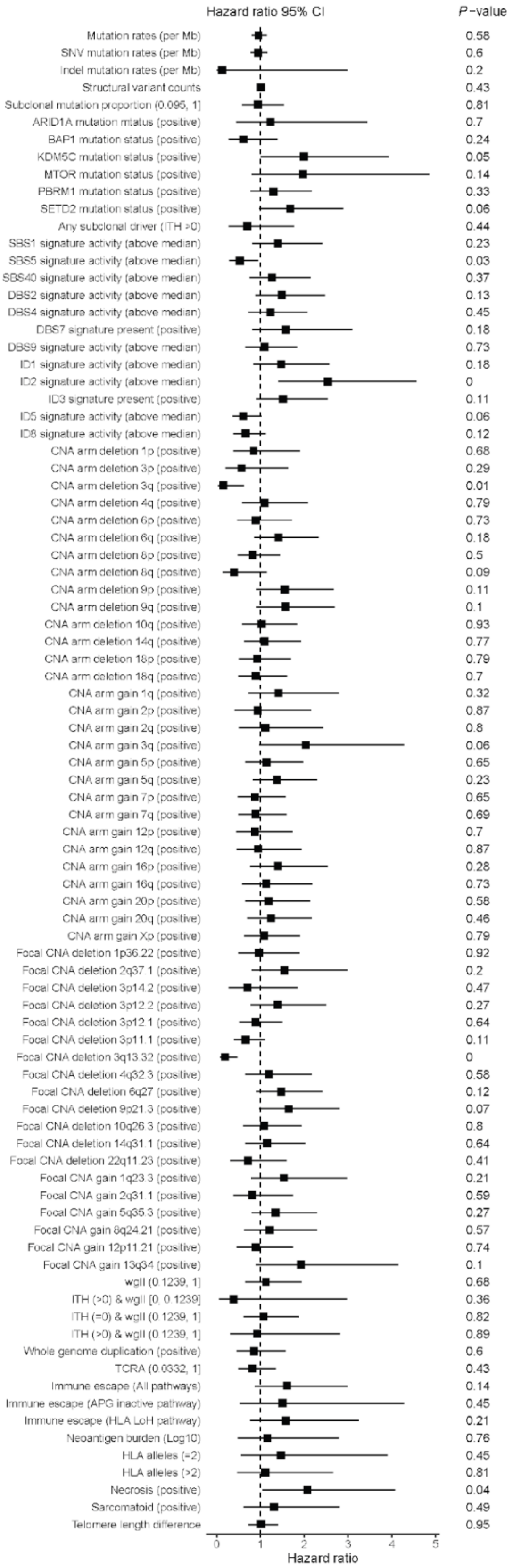

Supplementary Figure 15: Cox proportional hazards regression estimates for progression free survival. The hazard ratios are reported with their 95% confidence intervals and corresponding *P*-values. Sample sizes

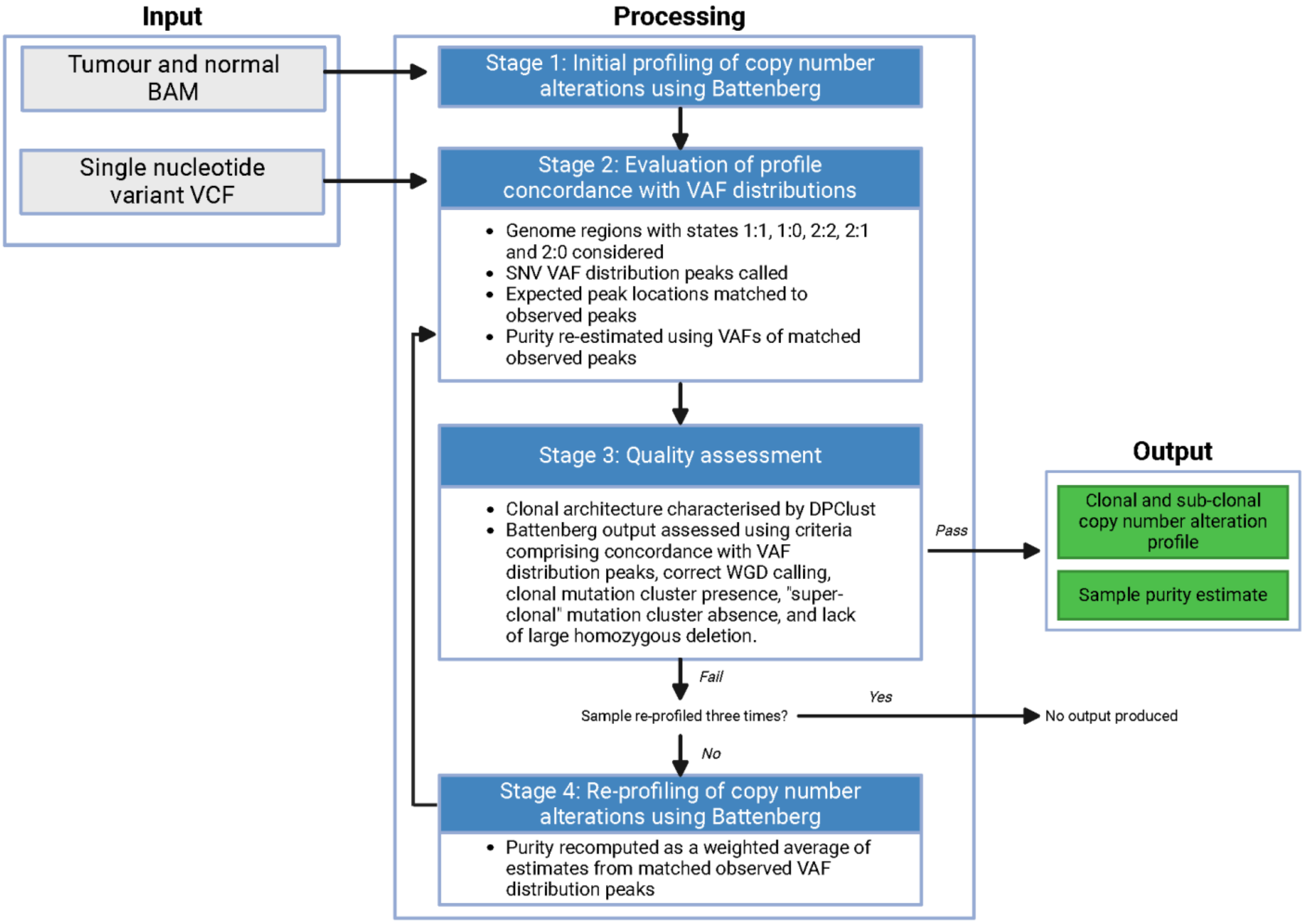

Supplementary Figure 16: Overview of copy-number-alteration-calling pipeline. BAM, binary sequence alignment map; SNV, single nucleotide variant; VAF, variant allele frequency; VCF, variant call format; WGD, whole genome duplication.

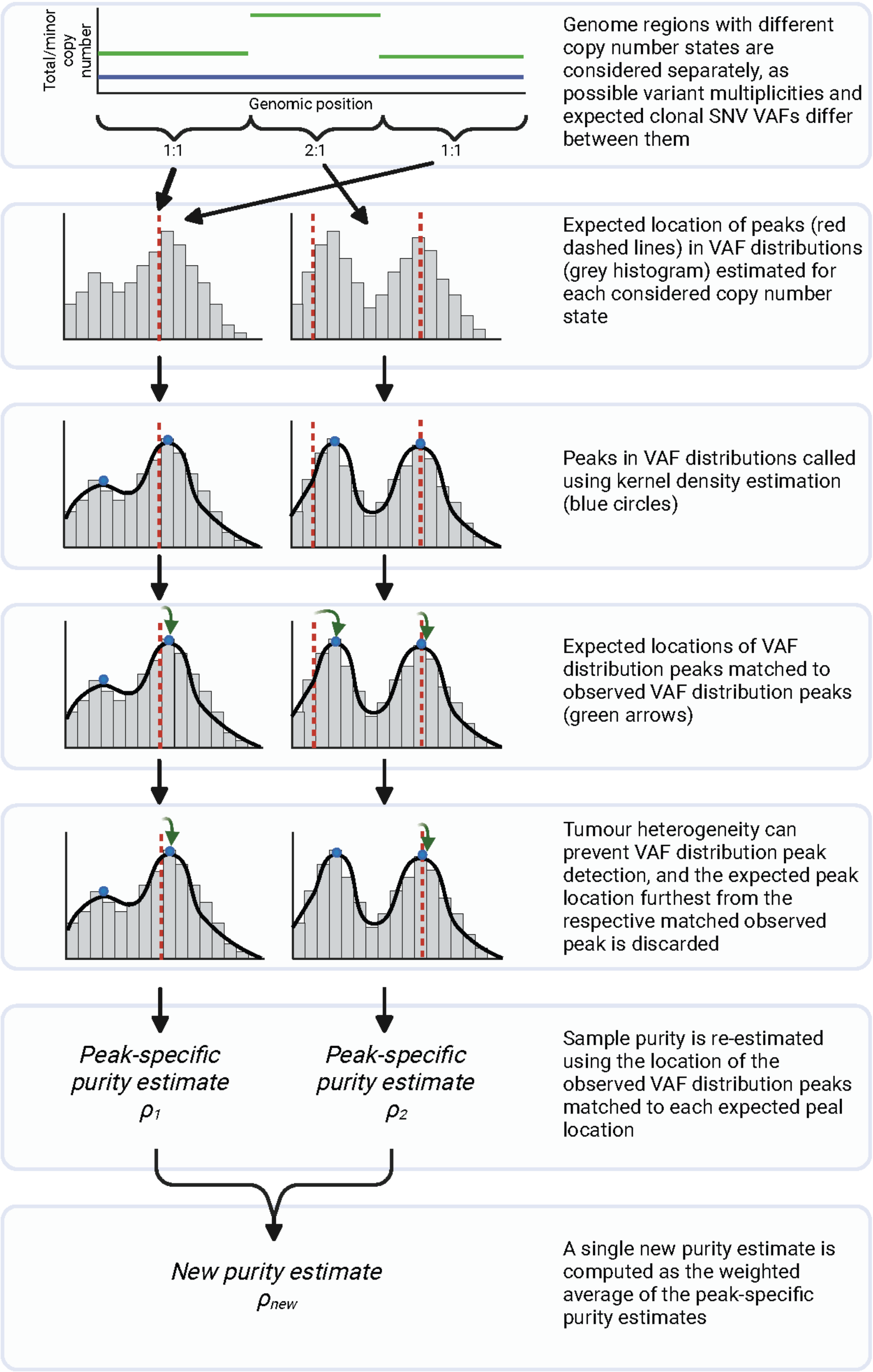

Supplementary Figure 17: Overview of stage two of the copy-number-alteration-calling pipeline. SNV, single nucleotide variant; VAF, variant allele frequency.

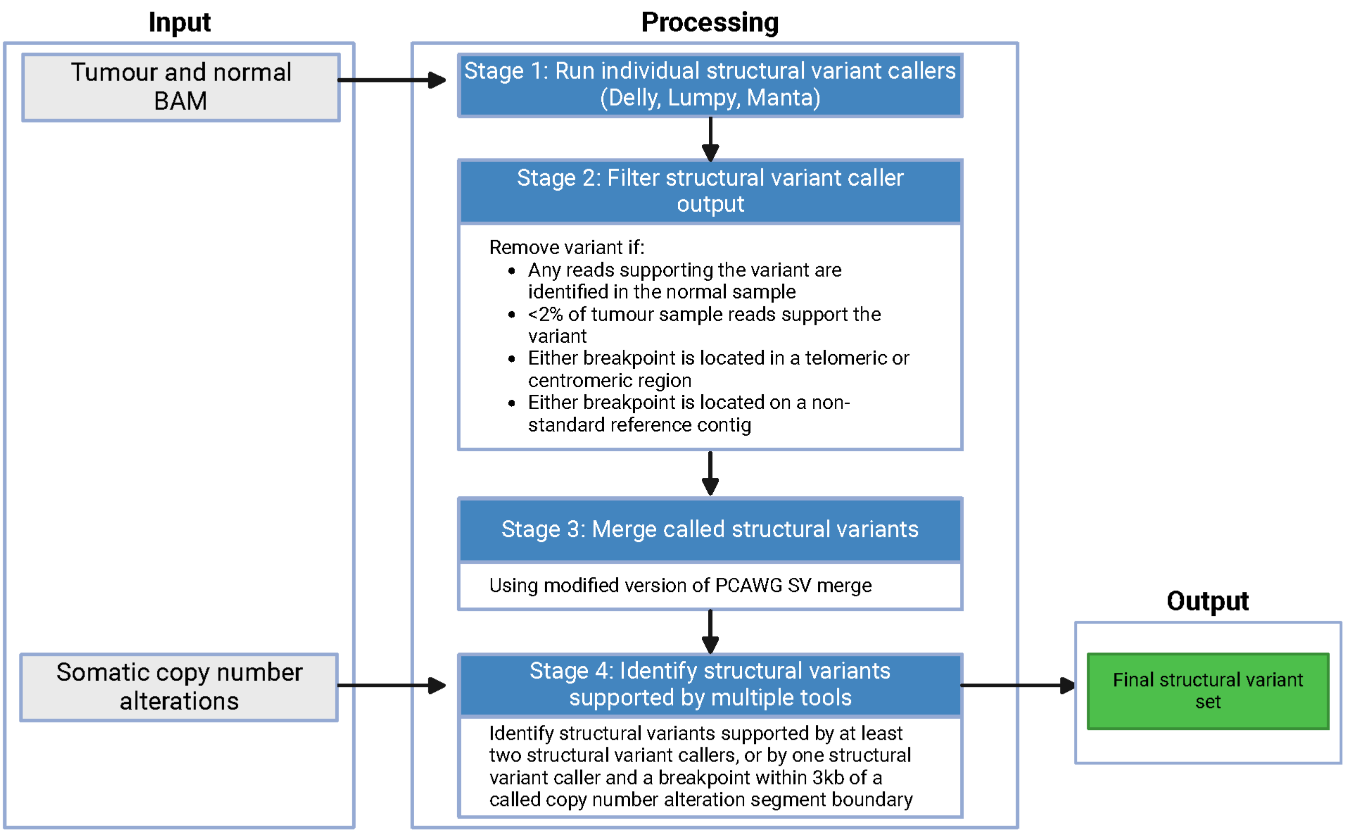

Supplementary Figure 18: Overview of structural-variant-calling pipeline. BAM, binary sequence alignment map; PCAWG, The Pan-Cancer Analysis of Whole Genomes; SV, structural variant.

SUPPLEMENTARY REFERENCES

1. Turnbull, C. Introducing whole-genome sequencing into routine cancer care: the Genomics England 100 000 Genomes Project. *Ann. Oncol.* **29**, 784–787 (2018).
2. Turnbull, C. *et al.* The 100 000 Genomes Project: bringing whole genome sequencing to the NHS. *BMJ* **361**, k1687 (2018).
3. Moch, H., Cubilla, A. L., Humphrey, P. A., Reuter, V. E. & Ulbright, T. M. The 2016 WHO Classification of Tumours of the Urinary System and Male Genital Organs—Part A: Renal, Penile, and Testicular Tumours. *Eur. Urol.* **70**, 93–105 (2016).
4. Raczy, C. *et al.* Isaac: ultra-fast whole-genome secondary analysis on Illumina sequencing platforms. *Bioinformatics* **29**, 2041–2043 (2013).
5. Saunders, C. T. *et al.* Strelka: accurate somatic small-variant calling from sequenced tumor–normal sample pairs. *Bioinformatics* **28**, 1811–1817 (2012).
6. Karczewski, K. J. *et al.* The mutational constraint spectrum quantified from variation in 141,456 humans. *Nature* **581**, 434–443 (2020).
7. Tate, J. G. *et al.* COSMIC: the Catalogue Of Somatic Mutations In Cancer. *Nucleic Acids Res.* **47**, D941–D947 (2018).
8. Benson, G. Tandem repeats finder: a program to analyze DNA sequences. *Nucleic Acids Res.* **27**, 573–580 (1999).
9. Jamal-Hanjani, M. *et al.* Tracking genomic cancer evolution for precision medicine: the lung TRACERx study. *PLoS Biol.* **12**, e1001906 (2014).
10. Cornish, A. J. *et al.* Reference bias in the Illumina Isaac aligner. *Bioinformatics* **36**, 4671–4672 (2020).
11. Salipante, S. J., Scroggins, S. M., Hampel, H. L., Turner, E. H. & Pritchard, C. C. Microsatellite Instability Detection by Next Generation Sequencing. *Clin. Chem.* **60**, 1192–1199 (2014).
12. Beier, S., Thiel, T., Münch, T., Scholz, U. & Mascher, M. MISA-web: a web server for microsatellite prediction. *Bioinformatics* **33**, 2583–2585 (2017).
13. Nik-Zainal, S. *et al.* The Life History of 21 Breast Cancers. *Cell* **149**, 994–1007 (2012).
14. Delaneau, O., Marchini, J. & Zagury, J.-F. A linear complexity phasing method for thousands of genomes. *Nat. Methods* **9**, 179–181 (2011).

15. Nilsen, G. *et al.* Copynumber: Efficient algorithms for single- and multi-track copy number segmentation. *BMC Genomics* **13**, 591 (2012).

16. Van Loo, P. *et al.* Allele-specific copy number analysis of tumors. *PNAS* **107**, 16910–16915 (2010).

17. Dentro, S. C., Wedge, D. C. & Van Loo, P. Principles of Reconstructing the Subclonal Architecture of Cancers. *Cold Spring Harb. Perspect. Med.* **7**, (2017).

18. Weber, C. M., Ramachandran, S. & Henikoff, S. Nucleosomes are context-specific, H2A.Z-modulated barriers to RNA polymerase. *Mol Cell* **53**, 819–830 (2014).

19. Rausch, T. *et al.* DELLY: structural variant discovery by integrated paired-end and split-read analysis. *Bioinformatics* **28**, i333–i339 (2012).

20. Layer, R. M., Chiang, C., Quinlan, A. R. & Hall, I. M. LUMPY: a probabilistic framework for structural variant discovery. *Genome Biol.* **15**, R84 (2014).

21. Chen, X. *et al.* Manta: rapid detection of structural variants and indels for germline and cancer sequencing applications. *Bioinformatics* **32**, 1220–1222 (2016).

22. Li, Y. *et al.* Patterns of somatic structural variation in human cancer genomes. *Nature* **578**, 112–121 (2020).

23. Chu, C. *et al.* Comprehensive identification of transposable element insertions using multiple sequencing technologies. *Nat. Commun.* **12**, 3836 (2021).

24. Rodriguez-Martin, B. *et al.* Pan-cancer analysis of whole genomes identifies driver rearrangements promoted by LINE-1 retrotransposition. *Nat. Genet.* **52**, 306–319 (2020).

25. Tubio, J. M. C. *et al.* Mobile DNA in cancer. Extensive transduction of nonrepetitive DNA mediated by L1 retrotransposition in cancer genomes. *Science* **345**, 1251343 (2014).

26. Gerstung, M. *et al.* The evolutionary history of 2,658 cancers. *Nature* **578**, 122–128 (2020).

27. Farmery, J. H. R., Smith, M. L., NIHR BioResource - Rare Diseases & Lynch, A. G. Telomerecat: A ploidy-agnostic method for estimating telomere length from whole genome sequencing data. *Sci. Rep.* **8**, 1300 (2018).

28. Dentro, S. C. *et al.* Characterizing genetic intra-tumor heterogeneity across 2,658 human cancer genomes. *Cell* **184**, 2239–2254.e39 (2021).

29. The ICGC/TCGA Pan-Cancer Analysis of Whole Genomes Consortium. Pan-cancer analysis of whole genomes. *Nature* **578**, 82–93 (2020).

30. McLaren, W. *et al.* The Ensembl Variant Effect Predictor. *Genome Biol.* **17**, 122 (2016).

31. Kircher, M. *et al.* A general framework for estimating the relative pathogenicity of human genetic variants. *Nat. Genet.* **46**, 310–315 (2014).

32. Rentzsch, P., Witten, D., Cooper, G. M., Shendure, J. & Kircher, M. CADD: predicting the deleteriousness of variants throughout the human genome. *Nucleic Acids Res.* **47**, D886–D894 (2019).

33. Rentzsch, P., Schubach, M., Shendure, J. & Kircher, M. CADD-Splice-improving genome-wide variant effect prediction using deep learning-derived splice scores. *Genome Med.* **13**, 31 (2021).

34. Zhang, X., Wakeling, M., Ware, J. & Whiffin, N. Annotating high-impact 5'untranslated region variants with the UTRannotator. *Bioinformatics* **37**, 1171–1173 (2021).

35. Martínez-Jiménez, F. *et al.* A compendium of mutational cancer driver genes. *Nat. Rev. Cancer* **20**, 555–572 (2020).

36. Priestley, P. *et al.* Pan-cancer whole-genome analyses of metastatic solid tumours. *Nature* **575**, 210–216 (2019).

37. Martincorena, I. *et al.* Universal Patterns of Selection in Cancer and Somatic Tissues. *Cell* **1731**, 1029–1041 (2017).

38. Mularoni, L., Sabarinathan, R., Deu-Pons, J., Gonzalez-Perez, A. & López-Bigas, N. OncodriveFML: a general framework to identify coding and non-coding regions with cancer driver mutations. *Genome Biol.* **17**, 128 (2016).

39. Arnedo-Pac, C., Mularoni, L., Muiños, F., Gonzalez-Perez, A. & Lopez-Bigas, N. OncodriveCLUSTL: a sequence-based clustering method to identify cancer drivers. *Bioinformatics* **35**, 5396 (2019).

40. Weghorn, D. & Sunyaev, S. Bayesian inference of negative and positive selection in human cancers. *Nat. Genet.* **49**, 1785–1788 (2017).

41. Dietlein, F. *et al.* Identification of cancer driver genes based on nucleotide context. *Nat. Genet.* **52**, 208–218 (2020).

42. Tokheim, C. *et al.* Exome-Scale Discovery of Hotspot Mutation Regions in Human Cancer Using 3D Protein Structure. *Cancer Res.* **76**, 3719–3731 (2016).

43. Burley, S. K. *et al.* RCSB Protein Data Bank: powerful new tools for exploring 3D structures of biological macromolecules for basic and applied research and education in fundamental biology, biomedicine,

biotechnology, bioengineering and energy sciences. *Nucleic Acids Res.* **49**, D437–D451 (2021).

44. Porta-Pardo, E. & Godzik, A. e-Driver: a novel method to identify protein regions driving cancer. *Bioinformatics* **30**, 3109–3114 (2014).
45. Martin, F. J. *et al.* Ensembl 2023. *Nucleic Acids Res.* **51**, D933–D941 (2023).
46. Sondka, Z. *et al.* The COSMIC Cancer Gene Census: describing genetic dysfunction across all human cancers. *Nat. Rev. Cancer* **18**, 696–705 (2018).
47. Lever, J., Zhao, E. Y., Grewal, J., Jones, M. R. & Jones, S. J. M. CancerMine: a literature-mined resource for drivers, oncogenes and tumor suppressors in cancer. *Nat. Methods* **16**, 505–507 (2019).
48. Bailey, M. H. *et al.* Comprehensive Characterization of Cancer Driver Genes and Mutations. *Cell* **173**, 371–385 (2018).
49. Cancer Genome Atlas Research Network. Comprehensive molecular characterization of clear cell renal cell carcinoma. *Nature* **499**, 43–49 (2013).
50. Turajlic, S. *et al.* Deterministic Evolutionary Trajectories Influence Primary Tumor Growth: TRACERx Renal. *Cell* **173**, 595–610.e11 (2018).
51. Clark, D. J. *et al.* Integrated Proteogenomic Characterization of Clear Cell Renal Cell Carcinoma. *Cell* **180**, 207 (2020).
52. Tsherniak, A. *et al.* Defining a cancer dependency map. *Cell* **170**, 564–576.e16 (2017).
53. Behan, F. M. *et al.* Prioritization of cancer therapeutic targets using CRISPR–Cas9 screens. *Nature* **568**, 511–516 (2019).
54. Weinstein, J. N., Collisson, E. A., Mills, G. B. & Shaw, K. R. The cancer genome atlas pan-cancer analysis project. *Nature* **45**, 1113–1120 (2013).
55. Lonsdale, J., Thomas, J., Salvatore, M., Phillips, R. & Lo, E. The genotype-tissue expression (GTEx) project. *Nature* **45**, 580–585 (2013).
56. Tang, Z. *et al.* GEPIA: a web server for cancer and normal gene expression profiling and interactive analyses. *Nucleic Acids Res.* **45**, W98–W102 (2017).
57. Cheng, J. *et al.* Accurate proteome-wide missense variant effect prediction with AlphaMissense. *Science* **381**, eadg7492 (2023).

58. Chakravarty, D. *et al.* OncoKB: A Precision Oncology Knowledge Base. *JCO Precis Oncol* **1**, 1–16 (2017).

59. Paczkowska, M. *et al.* Integrative pathway enrichment analysis of multivariate omics data. *Nat. Commun.* **11**, 735 (2020).

60. Subramanian, A. *et al.* Gene set enrichment analysis: a knowledge-based approach for interpreting genome-wide expression profiles. *Proc. Natl. Acad. Sci. U. S. A.* **102**, 15545–15550 (2005).

61. Liberzon, A. *et al.* The Molecular Signatures Database (MSigDB) hallmark gene set collection. *Cell Syst* **1**, 417–425 (2015).

62. Neph, S. *et al.* BEDOPS: high-performance genomic feature operations. *Bioinformatics* **28**, 1919–1920 (2012).

63. Zhu, H. *et al.* Candidate Cancer Driver Mutations in Distal Regulatory Elements and Long-Range Chromatin Interaction Networks. *Mol. Cell* **77**, 1307–1321.e10 (2020).

64. Rheinbay, E. *et al.* Analyses of non-coding somatic drivers in 2,658 cancer whole genomes. *Nature* **578**, 102–111 (2020).

65. Poole, W., Gibbs, D. L., Shmulevich, I., Bernard, B. & Knijnenburg, T. A. Combining dependent P-values with an empirical adaptation of Brown's method. *Bioinformatics* **32**, i430–i436 (2016).

66. Benjamini, Y. & Hochberg, Y. Controlling the false discovery rate: A practical and powerful approach to multiple testing. *J. R. Stat. Soc.* **57**, 289–300 (1995).

67. Mermel, C. H. *et al.* GISTIC2.0 facilitates sensitive and confident localization of the targets of focal somatic copy-number alteration in human cancers. *Genome Biology* **12**, R41 (2011).

68. Zack, T. I. *et al.* Pan-cancer patterns of somatic copy number alteration. *Nat Genet* **45**, 454–465 (2013).

69. Dale, R. K., Pedersen, B. S. & Quinlan, A. R. Pybedtools: a flexible Python library for manipulating genomic datasets and annotations. *Bioinformatics* **27**, 3423–3424 (2011).

70. Quinlan, A. R. & Hall, I. M. BEDTools: a flexible suite of utilities for comparing genomic features. *Bioinformatics* **26**, 841–842 (2010).

71. Cortés-Ciriano, I. *et al.* Comprehensive analysis of chromothripsis in 2,658 human cancers using whole-genome sequencing. *Nat. Genet.* **52**, 331–341 (2020).

72. Korbel, J. O. & Campbell, P. J. Criteria for inference of chromothripsis in cancer genomes. *Cell* **152**, 1226–1236 (2013).

73. Glodzik, D. *et al.* A somatic-mutational process recurrently duplicates germline susceptibility loci and tissue-specific super-enhancers in breast cancers. *Nat. Genet.* **49**, 341–348 (2017).
74. Haeussler, M. *et al.* The UCSC Genome Browser database: 2019 update. *Nucleic Acids Res.* **47**, D853–D858 (2019).
75. She, X. *et al.* Shotgun sequence assembly and recent segmental duplications within the human genome. *Nature* **431**, 927–930 (2004).
76. Weddington, N. *et al.* ReplicationDomain: a visualization tool and comparative database for genome-wide replication timing data. *BMC Bioinformatics* **9**, 530 (2008).
77. The ENCODE Project Consortium. An integrated encyclopedia of DNA elements in the human genome. *Nature* **489**, 57–74 (2012).
78. Luo, Y. *et al.* New developments on the Encyclopedia of DNA Elements (ENCODE) data portal. *Nucleic Acids Res.* **48**, D882–D889 (2020).
79. Bignell, G. J. *et al.* Signatures of mutation and selection in the cancer genome. *Nature* **463**, 893–898.
80. Barlow, J. H. *et al.* Identification of early replicating fragile sites that contribute to genome instability. *Cell* **152**, 620–632 (2013).
81. Beroukhim, R. *et al.* The landscape of somatic copy-number alteration across human cancers. *Nature* **463**, 899–905 (2010).
82. Le Tallec, B. *et al.* Common fragile site profiling in epithelial and erythroid cells reveals that most recurrent cancer deletions lie in fragile sites hosting large genes. *Cell Rep.* **4**, 420–428 (2013).
83. The ICGC/TCGA Pan-Cancer Analysis of Whole Genomes Consortium. Pan-cancer analysis of whole genomes. *Nature* **578**, 82–93 (2020).
84. Islam, S. M. A. *et al.* Uncovering novel mutational signatures by de novo extraction with SigProfilerExtractor. *Cell Genom* **2**, None (2022).
85. Everall, A. *et al.* Comprehensive repertoire of the chromosomal alteration and mutational signatures across 16 cancer types from 10,983 cancer patients. *medrxiv* 2023.06.07.23290970 (2023).
86. Davies, H. *et al.* HRDetect is a predictor of BRCA1 and BRCA2 deficiency based on mutational signatures. *Nat. Med.* **23**, 517–525 (2017).

87. Nik-Zainal, S. *et al.* Landscape of somatic mutations in 560 breast cancer whole-genome sequences. *Nature* **534**, 47–54 (2016).

88. Shukla, S. A. *et al.* Comprehensive analysis of cancer-associated somatic mutations in class I HLA genes. *Nat Biotechnol.* **33**, 1152–1158 (2015).

89. Hundal, J. *et al.* pVAC-Seq: A genome-guided in silico approach to identifying tumor neoantigens. *Genome Med.* **8**, 11 (2016).

90. McGranahan, N. *et al.* Allele-Specific HLA Loss and Immune Escape in Lung Cancer Evolution. *Cell* **171**, 1259–1271.e11 (2017).

91. Cornish, A. J. *et al.* Whole genome sequencing of 2,023 colorectal cancers reveals mutational landscapes, new driver genes and immune interactions. *bioRxiv* 2022.11.16.515599 (2022) doi:10.1101/2022.11.16.515599.

92. Kelly, A. & Trowsdale, J. Genetics of antigen processing and presentation. *Immunogenetics* **71**, 161–170 (2023).

93. Martínez-Jiménez, F. *et al.* Genetic immune escape landscape in primary and metastatic cancer. *Nat. Genet.* **55**, 820–831 (2023).
