# Supplementary material for: Whole genome sequencing refines stratification and therapy of patients with clear cell renal cell carcinoma
Source: Nat Commun. 2024 Jul 15;15:5935. doi: 10.1038/s41467-024-49692-1 (PMC11250826; doi:10.1038/s41467-024-49692-1)
Supplement: Supplementary file 3 — Description of Additional Supplementary Files [file 41467_2024_49692_MOESM3_ESM.pdf]

Description of Additional Supplementary Files

File Name: Supplementary Data 1

Description: Clinical characteristics of tested clear cell renal cell carcinoma subgroups.

File Name: Supplementary Data 2

Description: Mutational profile of clear cell renal cell carcinoma cohort.

File Name: Supplementary Data 3

Description: Germline mutations present in clear cell renal cell carcinoma cohort.

File Name: Supplementary Data 4

Description: Summary of coding driver genes for clear cell renal cell carcinoma.

File Name: Supplementary Data 5

Description: Driver gene co-occurrence analysis.

File Name: Supplementary Data 6

Description: Summary of non-coding driver regions for clear cell renal cell carcinoma.

File Name: Supplementary Data 7

Description: Summary of actionability of driver genes.

File Name: Supplementary Data 8

Description: Summary of recurrent arm level copy number alterations.

File Name: Supplementary Data 9

Description: Summary of recurrent focal copy number alteration regions.

File Name: Supplementary Data 10

Description: Summary of structural variant hotspot regions.

File Name: Supplementary Data 11

Description: Signature activity in clear cell renal cell carcinoma cohort.

File Name: Supplementary Data 12

Description: Univariate associations for molecular and clinical features.

File Name: Supplementary Data 13

Description: Logistic regression results on TCRA T-cell fraction.

File Name: Supplementary Data 14

Description: Negative binomial regression results on total neoantigen count per sample.

File Name: Supplementary Data 15

Description: Logistic regression results on immune escape pathways based on APG inactivating mutation, HLA LOH and any pathway.

File Name: Supplementary Data 16

Description: Negative binomial regression results on the number of HLA alleles.

File Name: Supplementary Data 17

Description: Cox proportional hazards regression results for baseline variables on overall survival, progression free survival and clear cell renal cell cancer specific survival.

File Name: Supplementary Data 18

Description: Cox proportional hazards regression results on overall survival and clear cell renal cell cancer specific survival.

File Name: Supplementary Data 19

Description: Cox proportional hazards regression results on progression free survival.

File Name: Supplementary Data 20

Description: Logistic regression results on necrosis.

File Name: Supplementary Data 21

Description: Logistic regression results on direct clinical benefit after immunotherapy.

File Name: Supplementary Data 22

Description: Landscape of survival analysis results for select variables.

File Name: Supplementary Data 23

Description: Number of clear cell renal cell carcinoma samples lost from quality control stages.

File Name: Supplementary Data 24

Description: Software and data used in this study.

File Name: Supplementary Data 25

Description: Classification of copy number alterations.
