# Supplementary material for: Whole genome sequencing refines stratification and therapy of patients with clear cell renal cell carcinoma
Source: Nat Commun. 2024 Jul 15;15:5935. doi: 10.1038/s41467-024-49692-1 (PMC11250826; doi:10.1038/s41467-024-49692-1)
Supplement: Supplementary file 5 — Reporting Summary [file 41467_2024_49692_MOESM5_ESM.pdf]

nature portfolio

Corresponding author(s): Richard Culliford
Charlie Mills
Sam Lawrence
Richard Houlston
Samra Turajlic

Last updated by author(s): May 22, 2024

Reporting Summary

Nature Portfolio wishes to improve the reproducibility of the work that we publish. This form provides structure for consistency and transparency in reporting. For further information on Nature Portfolio policies, see our Editorial Policies and the Editorial Policy Checklist.

Statistics

For all statistical analyses, confirm that the following items are present in the figure legend, table legend, main text, or Methods section.

n/a	Confirmed	
☐	☒	The exact sample size (*n*) for each experimental group/condition, given as a discrete number and unit of measurement
☐	☒	A statement on whether measurements were taken from distinct samples or whether the same sample was measured repeatedly
☐	☒	The statistical test(s) used AND whether they are one- or two-sided<br>*Only common tests should be described solely by name; describe more complex techniques in the Methods section.*
☐	☒	A description of all covariates tested
☐	☒	A description of any assumptions or corrections, such as tests of normality and adjustment for multiple comparisons
☐	☒	A full description of the statistical parameters including central tendency (e.g. means) or other basic estimates (e.g. regression coefficient) AND variation (e.g. standard deviation) or associated estimates of uncertainty (e.g. confidence intervals)
☐	☒	For null hypothesis testing, the test statistic (e.g. *F*, *t*, *r*) with confidence intervals, effect sizes, degrees of freedom and *P* value noted<br>*Give P values as exact values whenever suitable.*
☒	☐	For Bayesian analysis, information on the choice of priors and Markov chain Monte Carlo settings
☒	☐	For hierarchical and complex designs, identification of the appropriate level for tests and full reporting of outcomes
☒	☐	Estimates of effect sizes (e.g. Cohen's *d*, Pearson's *r*), indicating how they were calculated

Our web collection on statistics for biologists contains articles on many of the points above.

Software and code

Policy information about availability of computer code

Data collection

No software was used for data collection

Data analysis

Tumour-normal sample pairs were processed through 100,000 Genomes Project (100kGP) [GEL v14 data release] bioinformatics somatic variant analysis software pipeline. Information on sequencing and alignment can be found in the Genomics England Cancer Analysis Technical Information Document v11 (https://files.genomicsengland.co.uk/forms/Cancer-Analysis-Technical-Information-Document-v1-11-main.pdf)

After completion of the instructions given by the Data Availability statement, code to allow for reproducibility of results and figures are available in the Research Environment within the folder /re_gecip/shared_allGeCIPs/rculliford/ccRCC_landscape. Software packages, with corresponding download links and version numbers, can be found in Supplementary Data 24.

ActivePathways v1.1.1 (https://cran.r-project.org/web/packages/ActivePathways/index.html)
alleleCount-FixVAF (https://github.com/danchubb/alleleCount-FixVAF)
Battenberg v2.2.8 (https://github.com/Wedge-Oxford/battenberg)
BEDOPS v2.4.39 (https://github.com/bedops/bedops)
Bedtools v2.3.0 (https://github.com/arq5x/bedtools2)
CADD (https://github.com/kircherlab/CADD-scripts)
cBaSE (http://genetics.bwh.harvard.edu/wiki/sunyaevlab/cbase)
Ccube v1.0 (https://github.com/keyuan/ccube)
ClusterSV (https://github.com/cancerit/ClusterSV)
Delly v0.7.8 (https://github.com/dellytools/delly/releases/download/v0.7.9/delly_v0.7.9_linux_x86_64bit)

dNdSCV (https://github.com/im3sanger/dndscv)
DPClust v2.2.8 (https://github.com/Wedge-Oxford/dpclust)
FixVAF (https://github.com/danchubb/FixVAF)
GISTIC v2.0.2.3 (https://github.com/broadinstitute/gistic2)
HotMaps3D (https://github.com/KarchinLab/HotMAPS)
IntOGen (https://bitbucket.org/intogen/intogen-plus/src/master)
Isaac v03.16.02.19 (https://github.com/Illumina/Isaac3/releases/tag/iSAAC-03.16.02.19)
LiftOver (https://genome-store.ucsc.edu/)
LOHHLA (https://bitbucket.org/mcgranahanlab/lohhla/src/master/)
Lumpy v0.2.13 (https://github.com/arq5x/lumpy-sv/releases/download/0.2.13/lumpy-sv-v0.2.13.tar.gz)
Manta v0.28.0 (https://github.com/Illumina/manta/releases/download/v0.28.0/manta-0.28.0.release_src.tar.bz2)
MISA (https://webblast.ipk-gatersleben.de/misa/)
mSINGs (https://bitbucket.org/uwlabmed/msings/src/master/)
mutationTimeR v0.99.3 (https://github.com/gerstung-lab/MutationTimeR)
MutPanning (https://github.com/vanallenlab/MutPanningV2)
OncodriveClustl (https://bitbucket.org/bbglab/oncodriveclustl/src/master)
OncodriveFML (https://bitbucket.org/bbglab/oncodrivefml/src/master)
PCAWG SV merge (https://hub.docker.com/r/weischenfeldt/pcawg_sv_merge)
POLYSOLVER (https://github.com/jason-weirather/hla-polysolver)
pVACseq v3.1.2 (https://pvactools.readthedocs.io/en/latest/pvacseq.html)
pybedtools (https://daler.github.io/pybedtools/main.html)
Python v2.7, v3.6 (https://www.python.org/)
R v3.4.0, 4.10 (https://cran.ma.imperial.ac.uk/)
SHAPEIT2 v2.r904 (https://mathgen.stats.ox.ac.uk/genetics_software/shapeit/shapeit.html#download)
ShatterSeek v0.4 (https://github.com/parklab/ShatterSeek)
SigProfilerExtractor v1.1.4 (https://github.com/AlexandrovLab/SigProfilerExtractor/releases/tag/v1.1.4)
SIgProfilerMatrixGenerator v1.1.30 (https://github.com/AlexandrovLab/SigProfilerMatrixGenerator)
smRegions (https://bitbucket.org/bbglab/smregions/src/master)
Strelka v2.4.7 (https://github.com/Illumina/strelka/releases/tag/v2.4.7)
Survival v3.5-5 (https://cran.r-project.org/web/packages/survival/index.html)
Survminer v0.4.9 (https://cran.r-project.org/web/packages/survminer/index.html)
TelomereCat v3.3.0 (https://github.com/cancerit/telomerecat)
UTRannotator (https://github.com/ImperialCardioGenetics/UTRannotator)
VariantAnnotation v1.40.0 (https://bioconductor.org/packages/release/bioc/html/VariantAnnotation.html)
VEP (https://github.com/Ensembl/ensembl-vep)
xTea (https://github.com/parklab/xTea)

For manuscripts utilizing custom algorithms or software that are central to the research but not yet described in published literature, software must be made available to editors and reviewers. We strongly encourage code deposition in a community repository (e.g. GitHub). See the Nature Portfolio guidelines for submitting code & software for further information.

Data

Policy information about availability of data

All manuscripts must include a data availability statement. This statement should provide the following information, where applicable:

- Accession codes, unique identifiers, or web links for publicly available datasets
- A description of any restrictions on data availability
- For clinical datasets or third party data, please ensure that the statement adheres to our policy

The data supporting the findings of this study have been deposited in the National Genomic Research Library and can be accessed via the Genomics England Research Environment, a secure cloud workspace. The raw data, including patient profiles and corresponding genomic sequencing data, are only available under restricted access for patient privacy reasons. Access can be obtained by first applying to become a member of either the Genomics England Research Network (https://www.genomicsengland.co.uk/research/academic) or the Discovery Forum (industry partners https://www.genomicsengland.co.uk/research/research-environment). The process for joining the network is described at https://www.genomicsengland.co.uk/research/academic/join-gecip and consists of the following steps:
1. Your institution will need to sign a participation agreement available at https://files.genomicsengland.co.uk/documents/Genomics-England-GeCIP-Participation-Agreement-v2.0.pdf and email the signed version to gecip-help@genomicsengland.co.uk.
2. Once you have confirmed your institution is registered and have found a domain of interest, you can apply through the online form at https://www.genomicsengland.co.uk/research/academic/join-gecip where you can specify the reason for access and expected timeframe that you wish to have access. Once your Research Portal account is created you will be able to login and track your application.
3. Your application will be reviewed within 10 working days.
4. Your institution will validate your affiliation.
5. You will need to complete the online Information Governance training and will be granted access to the Research Environment within 2 days of passing the online training.

The processed clinical and genomic data applied to the investigation are available in the Research Environment within the folder /re_gecip/shared_allGeCIPs/rculliford/ccRCC_landscape. All other public/private datasets used in the study, including corresponding download links and version numbers, can be found in Supplementary Data 24.

AlphaMissense hg38 annotated missense mutations (Downloaded. August 3rd 2023, https://storage.cloud.google.com/dm_alphamissense/AlphaMissense_hg38.tsv.gz?_ga=2.165300023.-96076486.1696254104)
CADD v1.6 (https://cadd.gs.washington.edu/download)
CancerMine (Downloaded May 2022, http://bionlp.bcgsc.ca/cancermine/)
COSMIC Cancer Gene Census v92 (https://cancer.sanger.ac.uk/census)

COSMIC Mutation Actionability in Precision Oncology (https://cancer.sanger.ac.uk/actionability)
COSMIC Reference Mutational Signatures v3.2 (https://cancer.sanger.ac.uk/signatures/)
CHIP-Seq Histones H3K36me3 - Male adult (50 years) kidney tissue ENCODE4 v1.7.0 GRCh38-ENCAN946KXL (https://www.encodeproject.org/files/ENCFF191CJN/@@download/ENCFF191CJN.bigWig)
CHIP-Seq Histones H3K9me3 - Male adult (50 years) kidney tissue ENCODE4 v1.7.0 GRCh38-ENCAN127GOZ)https://www.encodeproject.org/files/ENCFF344RCN/@@download/ENCFF344RCN.bigWig)
DepMap CRISPI screens Last accessed 1st March 2024 (https://depmap.org/)
DNAse-seq - Female adult (47 years) kidney tissue ENCODE4 v3.0.0-alpha.2 GRCh38-ENCAN876VFO (https://www.encodeproject.org/files/ENCFF485CJZ/@@download/ENCFF485CJZ.bigWig)
Ensembl v101 (https://www.ensembl.org/index.html)
GnomAD v2.1 (https://gnomad.broadinstitute.org/downloads#v2-constraint)
GTEx Tissue Expression Profiles v7 (https://www.gtexportal.org/home/downloads/adult-gtex/overview)
Homo sapiens GRCh38Decoy reference assembly hg38(http://emea.support.illumina.com/sequencing/sequencing_software/igenome.html)
IntOGen Gene Annotations (Downloaded on 1st Febuary 2020, https://www.intogen.org/download?file=IntOGen-Cohorts-20200201.zip)
MSigDB Gene Sets v7.5.1 (https://www.gsea-msigdb.org/gsea/msigdb/human/collections.jsp)
OncoKB Knowledge Base v3.3 (https://www.oncokb.org/)
Protein Data Bank (PDB) (Download on March 2020, https://ftp.ebi.ac.uk/pub/databases/pdb_versioned/)
Replication Timing - Embryonic Kidney RT_HEK293_Embryonic Kidney_Int57383924(https://www2.replicationdomain.com/download_data.php?dataset=Int57383924&build=hg38)
Segmental Duplication Database (https://humanparalogy.gs.washington.edu)
TCGA kidney tumour-normal gene expression summary (Accessed February 2024, http://gepia.cancer-pku.cn/)
TCGA ccRCC cohort gene mutation frequency (Accessed February 2024, https://www.cbioportal.org/)
UCSC Genome Browser (https://hgdownload.soe.ucsc.edu/downloads.html)

Research involving human participants, their data, or biological material

Policy information about studies with human participants or human data. See also policy information about sex, gender (identity/presentation), and sexual orientation and race, ethnicity and racism.

Reporting on sex and gender	Patient sex was determined by generating XX of XY karyotypes from germline WGS data.<br><br>Frequencies of patient sex per stratified data are reported in Supplementary Table 1. Patient sex was included as a covariate in survival analysis and clinical correlations.
Reporting on race, ethnicity, or other socially relevant groupings	Race, ethnicity and other socially relevant grouping not reported.
Population characteristics	See above
Recruitment	Details of recruitment are provided in the "Ethics Statement" within the "Methods" section.
Ethics oversight	HRA Committee East of England - Cambridge South (REC Ref 14/EE/1112)

Note that full information on the approval of the study protocol must also be provided in the manuscript.

Field-specific reporting

Please select the one below that is the best fit for your research. If you are not sure, read the appropriate sections before making your selection.

☒ Life sciences ☐ Behavioural & social sciences ☐ Ecological, evolutionary & environmental sciences

For a reference copy of the document with all sections, see nature.com/documents/nr-reporting-summary-flat.pdf

Life sciences study design

All studies must disclose on these points even when the disclosure is negative.

Sample size	Details of sample sizes are provided in the Methods, Supplementary Methods, Figure 1 and Supplementary Data 1.
Data exclusions	Data exclusions are described in supplementary methods, and Supplementary Data 1 and 23.
Replication	Comparison of driver gene discovery, mutational signatures, mutation timing/ordering, recurrent CNAs/SVs,
Randomization	No randomisation applied
Blinding	No blinding applied

Reporting for specific materials, systems and methods

We require information from authors about some types of materials, experimental systems and methods used in many studies. Here, indicate whether each material, system or method listed is relevant to your study. If you are not sure if a list item applies to your research, read the appropriate section before selecting a response.

Materials & experimental systems

n/a	Involved in the study
☒	☐ Antibodies
☒	☐ Eukaryotic cell lines
☒	☐ Palaeontology and archaeology
☒	☐ Animals and other organisms
☐	☒ Clinical data
☒	☐ Dual use research of concern
☒	☐ Plants

Methods

n/a	Involved in the study
☒	☐ ChIP-seq
☒	☐ Flow cytometry
☒	☐ MRI-based neuroimaging

Clinical data

Policy information about clinical studies

All manuscripts should comply with the ICMJE guidelines for publication of clinical research and a completed CONSORT checklist must be included with all submissions.

Clinical trial registration	n/a
Study protocol	Details of the study protocol are provided by Genomics England (https://www.genomicsengland.co.uk/initiatives/100000-genomes-project/documentation)
Data collection	Data collection was carried out as detailed by Genomics England (https://www.genomicsengland.co.uk/initiatives/100000-genomes-project/documentation)
Outcomes	Reported outcomes include overall survival, progression free survival and adjusted/unadjusted clinical correlations.

Plants

Seed stocks	*Report on the source of all seed stocks or other plant material used. If applicable, state the seed stock centre and catalogue number. If plant specimens were collected from the field, describe the collection location, date and sampling procedures.*
Novel plant genotypes	*Describe the methods by which all novel plant genotypes were produced. This includes those generated by transgenic approaches, gene editing, chemical/radiation-based mutagenesis and hybridization. For transgenic lines, describe the transformation method, the number of independent lines analyzed and the generation upon which experiments were performed. For gene-edited lines, describe the editor used, the endogenous sequence targeted for editing, the targeting guide RNA sequence (if applicable) and how the editor was applied.*
Authentication	*Describe any authentication procedures for each seed stock used or novel genotype generated. Describe any experiments used to assess the effect of a mutation and, where applicable, how potential secondary effects (e.g. second site T-DNA insertions, mosiacism, off-target gene editing) were examined.*
